# Preclinical research and potential clinical application of traditional Chinese medicine in cancer treatment: take Astragalus and Dioscorea opposita as an example

**DOI:** 10.3389/fphar.2025.1662341

**Published:** 2025-09-25

**Authors:** Huizhu Jia, Yifan Qi, Hui Wang, Fengjiao Zhang, Jiarui Wu, Zhiqiang Zhang

**Affiliations:** ^1^ Henan University of Chinese Medicine, Zhengzhou, China; ^2^ Henan University of Animal Husbandry and Economy, Zhengzhou, China; ^3^ School of Chinese Materia Medica, Beijing University of Chinese Medicine, Beijing, China

**Keywords:** Astragalus membranaceus, Dioscorea opposita, therapeutic pair, cancer, immune microenvironment

## Abstract

**Background:**

Cancer is a complex and debilitating physical health disease that is important for social and individual health. Astragalus and Dioscorea opposita are two herbs that are traditionally used in folk medicine to treat various diseases, and they also play a role in immunizing and alleviating cancer.

**Purpose:**

This review aims to evaluate the anti-tumor potential of Astragalus and Dioscorea opposita by summarizing the active components and their known effects on tumor-related pathways, further illustrating the anti-tumor potential of traditional Chinese medicine. This study aims to explore the therapeutic effects of traditional Chinese medicines on different types of tumors.

**Methods:**

A comprehensive summary of the literature has been compiled, gathering information on the phytochemical characteristics, anti-tumor mechanisms, and the current status of both traditional Chinese and Western medicine relating to Astragalus and Dioscorea opposita. It demonstrates the previously established links between Astragalus and Dioscorea opposita and their anti-tumor properties.

**Results:**

The combined use of Astragalus and Dioscorea opposita enhances the immune regulatory effect through synergistic action, thereby improving the overall immune status of the body.

**Conclusion:**

This review provides a reference for experimental research in the treatment of tumors.

## Graphical Abstract

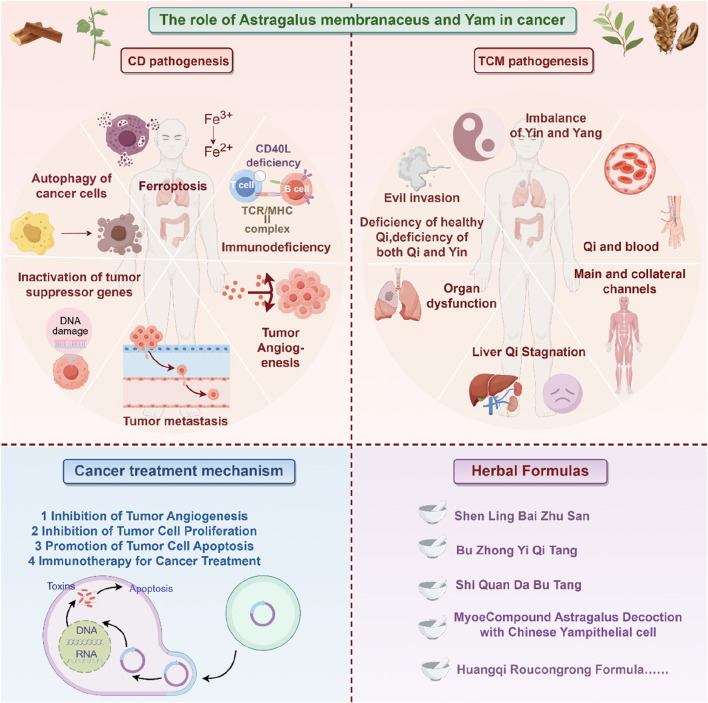

## 1 Introduction

Malignant tumors are diseases characterized by abnormal cell proliferation and are one of the leading causes of death globally. According to data from GLOBOCAN 2020, by 2040, the global number of new cancer cases is projected to reach 28.4 million, posing a serious threat to public health (Xu et al., 2009). Currently, major cancer treatments, including surgery, chemotherapy, radiation therapy, and molecular targeted therapy, show distinct limitations, such as the development of multi-drug resistance and severe side effects. Therefore, exploring new treatment methods and developing safe and effective new drugs is a pressing issue in the field of cancer prevention and treatment. In recent years, TCM has gained increasing attention due to its excellent anti-tumor efficacy and good safety profile, gradually becoming an important tool in cancer prevention and treatment.

### 1.1 Characteristics and experience of Chinese medicine in treating cancer

Traditional Chinese medicine treats cancer with a “holistic view” and “syndrome differentiation and treatment” as the core, emphasizing strengthening the body’s resistance and eliminating pathogens, and treating both the symptoms and the root causes. Its characteristics are as follows: regulating the balance of the body through multi-target actions such as invigorating qi and nourishing blood (such as Radix Astragali and Radix Angelicae Sinensis), clearing away heat and toxic materials (such as Herba Hedyotidis Diffusae and Herba Scutellariae Barbatae), softening and resolving hard mass (such as Prunellae Spica and Oyster), alleviating the toxic side effects such as bone marrow suppression and digestive tract reaction caused by radiotherapy and chemotherapy, and improving the quality of life indicators such as cancer fatigue and pain. Clinical experience shows that early intervention of traditional Chinese medicine can regulate the immune microenvironment, middle-term cooperation with surgical radiotherapy and chemotherapy can play a role in reducing toxicity and enhancing efficacy, and late intervention mainly focuses on strengthening the body’s resistance and strengthening the constitution to prolong the survival time with the tumor. For example, Sijunzi Decoction can improve spleen and stomach deficiency, and Biejiajian Pill can inhibit tumor angiogenesis.

Modern pharmacology has proved that Astragalus polysaccharides, ginsenosides, and other components can induce tumor cell apoptosis, but we should pay attention to avoid the abuse of “fighting poison with poison” and emphasize the coordinated, standardized treatment of traditional Chinese and Western medicine. Unlike the concept of Western medicine, traditional Chinese medicine emphasizes holistic treatment and the idea of “living with tumors,” aiming not only to target cancer cells and shrink tumors but also to improve the quality of life for cancer patients and extend their survival. Astragalus polysaccharides can reverse the multidrug resistance of tumor cells (as indicated by the downregulation of MDR1 gene expression), while Dioscorea opposita polysaccharides can reduce the toxicity of chemotherapy drugs. The combination of both can enhance the efficacy of chemotherapy and reduce side effects.

### 1.2 Application of traditional Chinese medicine pair in treating tumors

Traditional Chinese medicine has accumulated rich experience in the clinical application of cancer treatment, and its core lies in enhancing efficiency and reducing toxicity, and harmonizing yin and yang through drug compatibility. Astragalus membranaceus and Dioscorea opposita are often used as a therapeutic pair in TCM cancer treatment. A therapeutic pair, also known as a pair of medicines, refers to the combination of two commonly used, relatively fixed medicinal substances in clinical practice (Ayigaken et al., 2023). It follows the principles of TCM compatibility, reflecting the characteristics of TCM formulations and the philosophy of TCM diagnosis and treatment. Therapeutic pairs serve as the basic units for compound prescriptions. When used properly, they can achieve synergistic effects, while enhancing efficacy and reducing toxicity, as well as improving dissolution, promoting absorption, and remodeling metabolism (Qi, 2019). In-depth research on therapeutic pairs will help understand the scientific connotation of compound prescriptions. Astragalus membranaceus is the dried root of the leguminous plant Astragalus membranaceus or its variety Astragalus mongholicus, which acquires a sweet taste and slightly warm nature. Once it enters the lung and spleen meridians, Huangqi tonifies Qi and raises Yang, exerting beneficial effects on body metabolism (Yang et al., 2016). Dioscorea opposita is the tuber of the plant Dioscorea opposita in the Dioscoreaceae family, with a sweet taste, neutral nature, that functions in the lung, spleen, and kidney meridians. It tonifies the spleen and benefits the stomach, generates fluids and benefits the lungs, nourishes the kidneys and fills essence, and stops discharge and diarrhea (Yang et al., 2016). Both herbs are first mentioned in the Shennong Bencao Jing (Shennong Classic of Materia Medica) as superior herbs. Dioscorea opposita has a sweet taste, neutral nature, and enters the lung, spleen, and kidney meridians, tonifying the spleen, nourishing Yin, generating fluids, benefiting the lungs, and replenishing the five fatigue syndromes and seven injuries. Astragalus membranaceus has a sweet taste, slightly warm nature, and enters the spleen and lung meridians, tonifying Qi, raising Yang, and nourishing blood. The combination of these two herbs tonifies the spleen, strengthens the kidneys, nourishes Qi, and generates fluids. They act synergistically in balancing Yin and Yang, promoting spleen Qi, distributing essence to the lungs, and hydrating the body to relieve thirst. In the case of malignant tumors, blood stasis and meridian blockage occur, with the body’s righteous Qi being impaired, and both Qi and blood deficiency take place. Therefore, the strategy of tonifying Qi and promoting blood circulation is an effective approach in TCM for treating malignant tumors (Wu et al., 2018).

**FIGURE 1:**
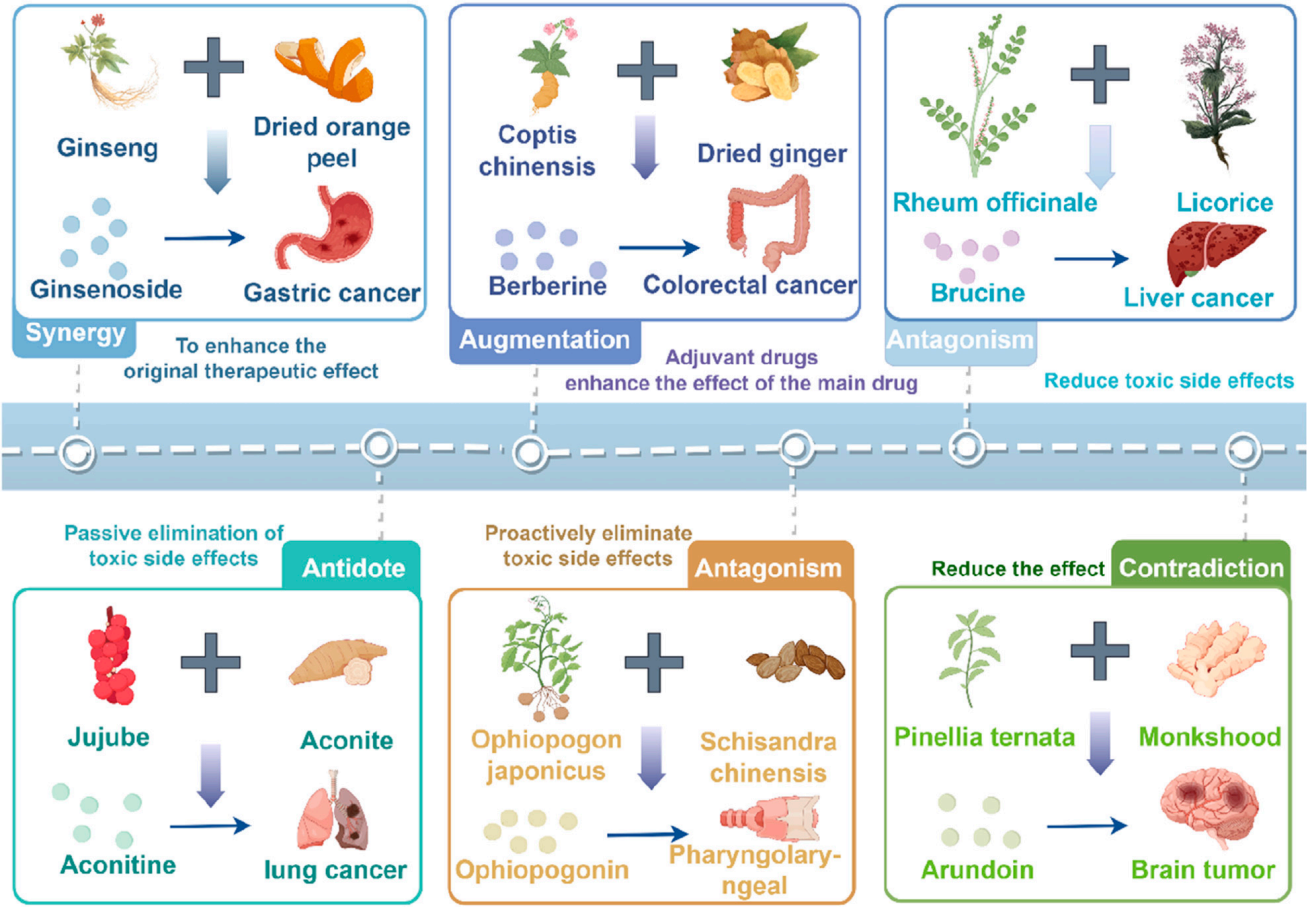
Commonly used traditional Chinese medicine herbal prescriptions for the treatment of tumors in clinical practice. The figure was drawn by Figdraw.

Astragalus membranaceus-Dioscorea opposita leverages the strengths of both herbs to address the pathological aspects of Qi deficiency and blood stasis in malignant tumors, reflecting the TCM formulation principles of “coordinating Qi and blood” and “treating both the deficiency and excess” (Wang, 2013). Both astragalus and Dioscorea opposita are included in the national catalog of medicine and food homology, emphasizing both medicinal values and edible functions on metabolic homeostasis. Astragalus is slightly warm in nature, holds the potential of replenishing qi and promoting yang, and is often used for the conditioning of individuals with physical weakness and low immunity; Dioscorea opposita is neutral in nature, strengthening the spleen and kidneys, and can help improve indigestion, diabetes, and chronic kidney disease. The combination of the two can harmonize Qi and Yin; for example, the traditional medicinal dish “astragalus and Dioscorea opposita porridge” can not only replenish the Qi within the lungs and spleen but also nourish the kidneys with Yin, making it suitable for individuals who are weak or in suboptimal health post-surgery. The combination of Astragalus membranaceus and Dioscorea opposita is not a random pairing, but a classical combination formed based on the principles of “functional complementarity and pathogenesis correspondence”. Its mechanism can be elucidated from the perspective of traditional Chinese medicine (TCM) theory: it achieves “simultaneous replenishment of Qi and Yin” and “simultaneous regulation of the Spleen and Kidney”, which aligns with the core pathogenesis of cancer characterized by “deficiency of healthy Qi” in TCM. Modern studies indicate that astragalus polysaccharides and Dioscorea opposita polysaccharides synergistically enhance immune regulation and antioxidant capacity, with their extracts displaying significant effects in combating fatigue and lowering blood sugar, among other areas. In daily life, these can be prepared as soups, tea, or incorporated into dishes (such as stewed chicken with astragalus or blueberry Dioscorea opposita puree), but it is important to be cautious: those with real heat syndrome should avoid astragalus, and those with excessive dampness and abdominal bloating should exercise caution with Dioscorea opposita. It is recommended to use them under the guidance of a traditional Chinese medicine practitioner. Application of the Traditional Chinese medicine pair in Treating Tumors is shown in Figure 1. Contains Classic Prescriptions with Astragalus and Dioscorea opposita are shown in Table 1.

**TABLE 1:** Contains classic prescriptions with Astragalus and Dioscorea opposita.

Formula name	Major composition	Original source	Refences
Wen Hua Tang	Astragalus, Chinese Dioscorea opposita, Rehmannia, Schisandra, Cowherb seed, etc.	*Encyclopedia of Chinese Medical Secret Formulas*	Zhang and Hu (2010)
Bu Yi Xiao Ai Tang	Astragalus, Chinese Dioscorea opposita, Schisandra, Ginseng, Prepared Rehmannia, etc.	Ming Dynasty physician Li Shizhong	Zhang and Hu (2010)
Xiao Liu San Jie Tang	Astragalus, Chinese Dioscorea opposita, White Atractylodes, Poria, Chuanxiong, etc.		Zhang and Hu (2010)
Huang Qi Zhi Shi Tang	Astragalus, Chinese Dioscorea opposita, Fleeceflower root, Codonopsis, Solomon’s seal, etc.		Zhang and Hu (2010)
Shen Qi Di Huang Tang	Ginseng, Astragalus, Rehmannia, Chinese Dioscorea opposita	Minfukang	Zhang and Hu (2010)
Kang Ai Tang	Chinese Dioscorea opposita, Astragalus, Tremella, Mulberry leaf, Prepared Rehmannia, etc.		Zhang and Hu (2010)
Lung Cancer Qi-Yin Deficiency Formula	Astragalus, Chinese Dioscorea opposita, Glehnia, Ophiopogon, Houttuynia, etc.		Zhang and Hu (2010)
Da Xu Ming Tang	Astragalus, Chinese Dioscorea opposita, Codonopsis, White Atractylodes, Cinnamon twig, etc.		Zhang and Hu (2010)
Modified Bu Zhong Yi Qi Tang	Raw Astragalus, White Atractylodes, Codonopsis, Poria, Chinese Dioscorea opposita, Chicken gizzard, Bupleurum, Licorice, Angelica, Tangerine peel, Pinellia, Bitter orange, etc.	Liu Yaxian’s medical records	Xue et al. (2018)
Shu Yu Wan	Chinese Dioscorea opposita, Angelica, Cinnamon twig, Fermented leaven, Rehmannia, Soybean sprout, Licorice, Ginseng	*Synopsis of the Golden Chamber*	Xue et al. (2018)
Huang Qi Kang Ai Tang	Raw Astragalus, Oldenlandia, Coptis, Scutellaria		Xue et al. (2018)
Sheng Xue Tang	Astragalus, Spatholobus, Wolfberry, Dodder seed, Solomon’s seal, etc.		Xue et al. (2018)
Zi Yi Yin	Raw Astragalus, Raw Chinese Dioscorea opposita, Raw Rehmannia, Cornus, Pork pancreas	*Records of Traditional Chinese and Western Medicine in Combination*	Xue et al. (2018)
Li Chong Tang	Raw Astragalus, Raw Chinese Dioscorea opposita, Codonopsis, White Atractylodes, Anemarrhena, Burreed tuber, Zedoary, etc.	*Records of Traditional Chinese and Western Medicine in Combination*	Ju et al. (2014)
Shi Quan Yu Zhen Tang	Astragalus, Chinese Dioscorea opposita, Wild Codonopsis, Anemarrhena, Scrophularia, Dragon bone, Oyster shell, etc.	*Records of Traditional Chinese and Western Medicine in Combination*	Ju et al. (2014)
Yu Ye Tang	Astragalus, Chinese Dioscorea opposita, Anemarrhena, Trichosanthes root, Kudzu root, Schisandra, etc.	*Records of Traditional Chinese and Western Medicine in Combination*	Ju et al. (2014)
Shen Qi Di Huang Tang	Astragalus, Chinese Dioscorea opposita, Ginseng, Rehmannia, Poria, etc.		Ju et al. (2014)
Pi Shen Liang Zhu Wan	Astragalus, Chinese Dioscorea opposita, Codonopsis, White Atractylodes, Prepared Rehmannia, Eucommia, etc.		Ju et al. (2014)
Fang Ji Huang Qi Tang	Astragalus with Stephania root, White Atractylodes, Licorice, Ginger, Jujube	*Synopsis of the Golden Chamber*	Fang et al. (2009)
Qi Huai Tang		*Shi Jinmo’s Clinical Experience Collection*	Fang et al. (2009)
Huang Qi Compound	Raw Astragalus, Chinese Dioscorea opposita, White Atractylodes, Tangerine peel, Raw Rehmannia, Poria	*Ancient and Modern Prescriptions*	Fang et al. (2009)
Huang Qi Shan Yao Tea	Astragalus, Chinese Dioscorea opposita, Flower tea	*Tea and Health*	Fang et al. (2009)
Shen Rou Yang Zhen Tang	Four Gentlemen Decoction plus Astragalus, Chinese Dioscorea opposita, White peony, Lotus seed, Ophiopogon, Schisandra		Fang et al. (2009)
Huang Qi Shi Hu Wine	Dendrobium, Astragalus, Chinese Dioscorea opposita, Codonopsis, Saposhnikovia, Salvia	*Sheng Ji Zong Lu*	Fang et al. (2009)
Huang Qi Dang Gui Bu Xue Tang	Astragalus, Angelica, Peanut, Jujube	A folk anti-cancer remedy, known as “Top Anti-Cancer Soup”	Fang et al. (2009)
Astragalus with Oldenlandia	Astragalus, Oldenlandia, Scutellaria, Wolfberry	Clinical TCM experience	Fang et al. (2009)
Cancer Six-Ingredient Decoction	Astragalus, Angelica, White peony, Licorice, Tangerine peel, Longan	Common clinical formula for tumors	Fang et al. (2009)
Rectal Cancer Decoction	Astragalus, Raw Sanguisorba, Angelica, White peony, Honeysuckle, Patrinia, Sophora charcoal	TCM formula for colorectal cancer	Fang et al. (2009)
Stomach Cancer Adjuvant	Astragalus, Ginseng, Wolfberry	*Imperial Medical Formulas*	Fang et al. (2009)
Nei Bu Huang Qi Tang	Astragalus, Angelica, Rehmannia, Ophiopogon, Ginger, Jujube, etc.	*Supplement to the Thousand Gold Formulas*	Fang et al. (2009)
Professor Yu Rencun’s Formula	Astragalus, Lingzhi, Codonopsis, etc.	Clinical experience of Prof. Yu Rencun, Beijing TCM Hospital	Fang et al. (2009)
Qi-Blood Dual-Tonic Anti-Cancer Formula	Glehnia, Ophiopogon, Schisandra, Fragrant seal, Chinese Dioscorea opposita, Dendrobium, Jujube seed, Biota seed, etc.	*Complete Collection of Anti-Cancer Chinese Formulas*	Tsukui et al. (1999)
Spleen-Kidney Strengthening Formula	Chinese Dioscorea opposita, Astragalus, Angelica, White peony, Licorice, Tangerine peel, Longan	Common clinical formula for tumors	Tsukui et al. (1999)
Dry-Dampness Balancing Formula	Chinese Dioscorea opposita, Poria, Alisma	Zhang Xichun’s *Records of Traditional Chinese and Western Medicine in Combination*	Tsukui et al. (1999)
Chinese Dioscorea opposita External Application	Fresh Chinese Dioscorea opposita, Chuanxiong powder, Sugar	*Ben Jing Feng Yuan*	Tsukui et al. (1999)
Bladder Cancer Folk Remedy	Raw Rehmannia, Chinese Dioscorea opposita, Cornus, Poria, Polyporus, Lithospermum, Aloe	Expert opinion (2020)	Nagai et al. (2006)
Esophageal Cancer Formula	Coix seed, Chinese Dioscorea opposita, Hyacinth bean, Cardamom, Tangerine peel, Pinellia, Inula, Hematite	Expert opinion (2020)	Nagai et al. (2006)
Lung Cancer Folk Remedy	Prunella, Kelp, Sargassum, Oyster shell	Expert opinion (2020)	Nagai et al. (2006)

## 2 Chemical composition of Astragalus membranaceus

### 2.1 Flavonoid components

**FIGURE 2:**
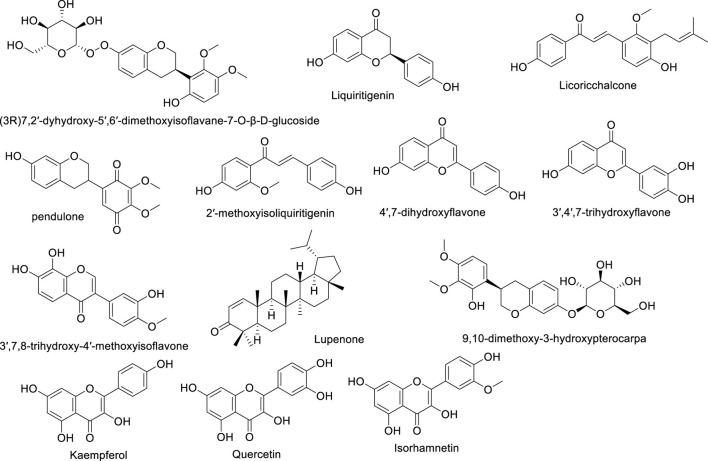
The structural formula of Astragalus flavonoids.

**FIGURE 3:**
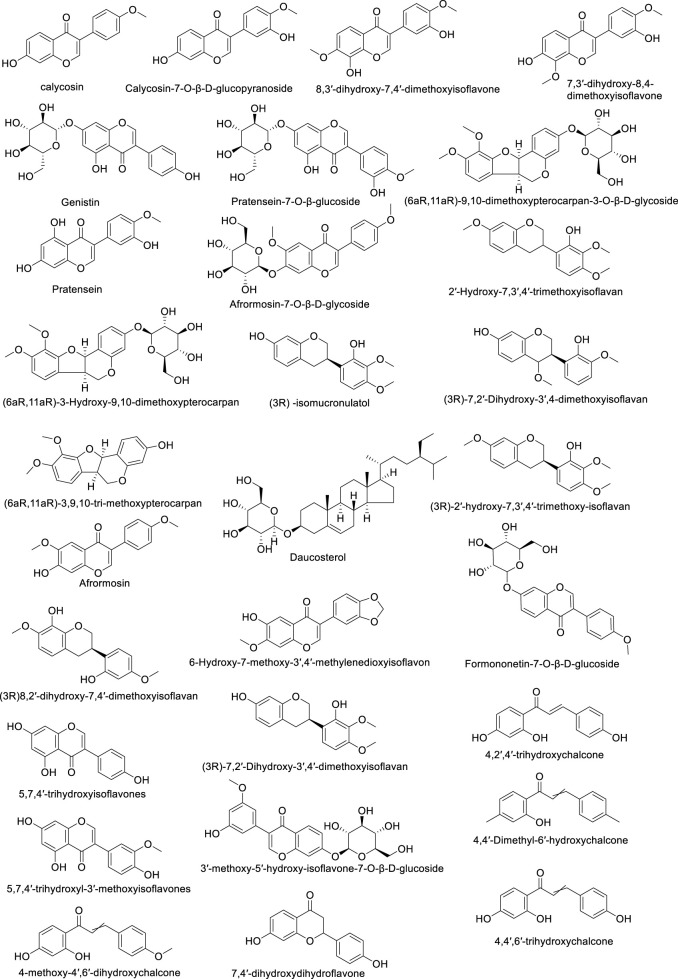
The structural formula of Astragalus flavonoids.

Astragalus flavonoids are one of the important secondary metabolites in Astragalus membranaceus, exhibiting pharmacological activities such as immune-enhancing and antioxidant effects (Liao et al., 2017). They are capable of complexing free radicals, reducing inflammation, and thus play a crucial role in maintaining human health. The flavonoid components of Astragalus membranaceus are summarized in Table 2. The structural formula of Astragalus flavonoids is shown in Figures 2, 3.

**TABLE 2:** Flavonoid components of Astragalus membranaceus.

Serial no.	Compound names	References
1	Formononetin	(Song and Sheng, 1997)
2	Calycosin	(Song and Sheng, 1997)
3	Calycosin-7-O-β-D-glucopyranoside	(Song and Sheng, 1997)
4	Odoratin-7-O-β-glucopyranoside	(Song and Sheng, 1997)
5	8,3′-dihydroxy-7,4′-dimethoxyisoflavone	(Song and Sheng, 1997)
6	7,3′-dihydroxy-8,4-dimethoxyisoflavone	(Song and Sheng, 1997)
7	Genistin	(Wen, 2006)
8	Pratensein-7-O-β-glucoside	(Wen, 2006)
9	(6aR,11aR)-9,10-dimethoxypterocarpan-3-O-β-D-glycoside	(Zhang et al., 2007)
10	Pratensein	(Zhang et al., 2007)
11	Afrormosin-7-O-β-D-glycoside	(Zhang et al., 2007)
12	Calycosin7-O-β-D-glycoside-6ʺ-O-acetat	(Zhang et al., 2007)
13	2′-Hydroxy-7,3′,4′-trimethoxyisoflavan	(Zhang et al., 2007)
14	Formononetin 7-O-β-D-glycoside-6ʺ-O-acetate	(Zhang et al., 2007)
15	Formosin7-O-β-D-glycoside-6ʺ-O-malonate	(Zhang et al., 2007)
16	Calycosin7-O-β-D-glycoside-6ʺ-O-malonate	(Zhang et al., 2007)
17	Formononetin 7-O-β-D-glycoside-6ʺ-O-malonate	(Zhang et al., 2007)
18	9,10-Dimethoxypterocarpan-3-O-β-D-glycoside	(Zhang et al., 2007)
19	2′-Hydroxy-3′,4′-dimethoxyisoflavan-7-O-β-D-glycoside	(Zhang et al., 2007)
20	(6aR,11aR)-3-Hydroxy-9,10-dimethoxypterocarpan	(Zhang et al., 2007)
21	(3R)-7,2′-Dihydroxy-3′,4-dimethoxyisoflavan	(Zhang et al., 2007)
22	Astrapterocarpanglucoside-6ʺ-O-malonate	(Zhang et al., 2007)
23	Astraisoflavanglucoside-6ʺ-O-malonate	(Lin et al., 2000)
24	(3R) –isomucronulatol	(Pei et al., 2008)
25	(3R) -7-O-β-glucoside-isomucronulatol	(Subarnas et al., 1991)
26	Isomucronulatol 7,2′-di-O-glcoside	(Subarnas et al., 1991)
27	5′-hydroxyisomucronulatol 2′,5′-di-O-glcoside	(Subarnas et al., 1991)
28	7-O-methylisomucronulatol	(Subarnas et al., 1991)
29	3,9-di-O-methylnissolin	(Subarnas et al., 1991)
30	Isomucronulatol 7-O-glucoside	(Subarnas et al., 1991)
31	(3R) −8,2′-di-OH-7,4′-dimethoxyisoflavane	(Subarnas et al., 1991)
32	(3R)-7,2′,3′-trihydroxy-4′-methoxyisoflavan	(Subarnas et al., 1991)
33	(6aR,11aR)-3,9-dimethoxy-10-hydroxypterocarpan	(Wible et al., 2009)
34	(6aR,11aR)-3,9,10-tri-methoxypterocarpan	(Wible et al., 2009)
35	(3R)-2′-hydroxy-7,3′,4′-trimethoxy-isoflavan	(Wible et al., 2009)
36	Daucosterol	(Wible et al., 2009)
37	Afrormosin	(Wible et al., 2009)
38	Formononetin-7-O-β-D-glucoside	(Wible et al., 2009)
39	(3R)8,2′-dihydroxy-7,4′-dimethoxyisoflavan	(Wible et al., 2009)
40	6-Hydroxy-7-methoxy-3′,4′-methylenedioxyisoflavone	(Wible et al., 2009)
41	5′,7-di-OH-3′-methoxyisoflavone	(Lenssen et al., 1994)
42	(3R)-2′-hydroxy-3′,4′-dimethoxyisoflavan-7-O-β-D-glucoside	(Lenssen et al., 1994)
43	5′-hydroxy-3′-methoxyisoflavone-7-O-β-D-glucoside	(Lenssen et al., 1994)
44	(3R)-7,2′-Dihydroxy-3′,4′-dimethoxyisoflavan	(Lenssen et al., 1994)
45	5,7,4′-trihydroxyisoflavones	(Molodavkin et al., 2000)
46	4,2′,4′-trihydroxychalcone	(Molodavkin et al., 2000)
47	(3R)-8,2′-diydroxy-7,4-dimethoxy-isoflavone	(Molodavkin et al., 2000)
48	7,2′-dyhydroxy-3′,4′-dimethoxy-isoflavane-3-O-β-D-glucoside	(Molodavkin et al., 2000)
49	6ʺ-O-acetyl-formononetin	(Yan et al., 2010)
50	6ʺ-O-acetyl-(3R)-7,2′-dyhydroxy-3′,4′-dimethoxy-isoflavane-7-O-β-D-glucoside	(Yan et al., 2010)
51	6ʺ-O-acetyl-(6aR,11aR)-3-hydroxy-9,10-dimethoxy-rosealtane-3-O-β-D-glucoside	(Yan et al., 2010)
52	5,7-dyhydroxy-4′-methoxyisoflavones-7-O-β-D-glucoside	(Yan et al., 2010)
53	5,7,4′-trihydroxyl-3′-methoxyisoflavones	(Yan et al., 2010)
54	(−)-methylnissolin 3-O-β-D-(6′-acetyl)-glucoside	(Měsíek and Soják, 1969)
55	(−)-methylnissolin 3-O-β-D-(Xiao-Juan)-glucoside	(Měsíek and Soják, 1969)
56	(−)-methylnissolin 3-O-β-D-glucoside	(Měsíek and Soják, 1969)
57	3′-methoxy-5′-hydroxy-isoflavone-7-O-β-D-glucoside	(Měsíek and Soják, 1969)
58	4,4′-Dimethyl-6′-hydroxychalcone	(Kim et al., 2010)
59	4-methoxy-4′,6′-dihydroxychalcone	(Mohammed et al., 2007)
60	7,4′-dihydroxydihydroflavone	(Mohammed et al., 2007)
61	4,4′,6′-trihydroxychalcone	(Mohammed et al., 2007)
62	4′-hydroxydihydroflavone-7-O-β-D-glucoside	(Mohammed et al., 2007)
63	3,2′-dihydroxy-3′,4′-dimethoxyisoflavane-7-O-β-D-glucoside	(Mohammed et al., 2007)
64	(3R)7,2′-dyhydroxy-5′,6′-dimethoxyisoflavane-7-O-β-D-glucoside	(Xie et al., 1997)
65	Liquiritigenin	(Xie et al., 1997)
66	Pendulone	(Xie et al., 1997)
67	Licoricchalcone	(Xie et al., 1997)
68	Licorice chalcone	(Xie et al., 1997)
69	2′-methoxyisoliquiritigenin	(Xie et al., 1997)
70	4′,7-dihydroxyflavone	(Xie et al., 1997)
71	3′,4′,7-trihydroxyflavone	(Xie et al., 1997)
72	3′,7,8-trihydroxy-4′-methoxyisoflavone	(Xie et al., 1997)
73	Lupenone	(Xie et al., 1997)
74	9,10-dimethoxy-3-hydroxypterocarpa	(Xie et al., 1997)
75	10-(6αR,11αR)-3,9-dimethoxy-10-hydroxy-pterocarpan	(Xie et al., 1997)
76	Kaempferol	(Xie et al., 1997)
77	Quercetin	(Xie et al., 1997)
78	Isorhamnetin	(Xie et al., 1997)
79	Rhamnocitrin	(Xie et al., 1997)
80	Kumatakenin	(Xie et al., 1997)
81	Glycitein	(Xie et al., 1997)
82	Glycitin	(Xie et al., 1997)
83	Rhamnocitrin-3-O-β-D-glucoside	(Xie et al., 1997)
84	Rhamnocitrin-3-O-β-neohesperidoside	(Xie et al., 1997)
85	Rhamnocitrin-3-O-β-D-glucopyranoside(1→2″)-β-D-apiofuranosyl	(Xie et al., 1997)
86	Complanatuside	(Xie et al., 1997)
87	Tiliroside	(Xie et al., 1997)

### 2.2 Saponin components

**FIGURE 4:**
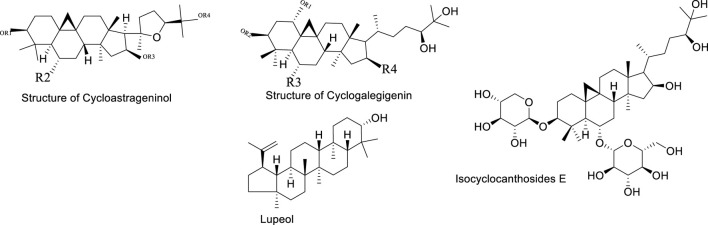
The structural formula of Astragalus membranaceus.

Although the saponin compounds in Astragalus membranaceus are present in relatively low concentrations, they are diverse in types. Astragalus saponins belong to the group of triterpenoid saponins and their derivatives. The structure of these compounds is primarily composed of a sapogenin core and varying numbers and types of sugar moieties (Li and Yang, 2005). The saponin components of Astragalus membranaceus are summarized in Tables 3–5. The structural formula of Astragalus membranaceus is shown in Figure 4.

**TABLE 3:** Saponin components of astragalus membranaceus.

Serial no.	Compound name	References
1	Cyclocanthoside A	(Mohammed et al., 2007)
2	Isoastragaloside Ⅳ	(Mohammed et al., 2007)
3	Cyclocanthoside E	(Mohammed et al., 2007)
4	Astragaloside Ⅶ	(Mohammed et al., 2007)
5	Astragaloside Ⅲ	(Mohammed et al., 2007)
6	Astragaloside Ⅵ	(Mohammed et al., 2007)
7	Astragaloside Ⅳ	(Mohammed et al., 2007)
8	Isoastragaloside Ⅱ	(Gao et al., 2020)
9	Astragaloside Ⅱ	(Gao et al., 2020)
10	Astragaloside Ⅲ	(Gao et al., 2020)
11	Isoastragaloside Ⅰ	(Gao et al., 2020)
12	Brachyoside B	(Gao et al., 2020)
13	Cycloaraloside A	(Gao et al., 2020)
14	Astragaloside Ⅰ	(Ya and Mei, 2005)
15	Astragaloside Ⅱ	(Ya and Mei, 2005)
16	Astragaloside Ⅳ	(Yan et al., 2010)
17	Astragaloside Ⅴ	(Yan et al., 2010)
18	Astragaloside Ⅳ	(Yan et al., 2010)
19	Acetytastragaloside	(Xie et al., 1997)
20	Agroastragalosides Ⅲ	(Zhou et al., 1995)
21	Agroastragalosides Ⅳ	(Zhou et al., 1995)
22	Astramembranoside B	(Ju et al., 2008)
23	Isoastragaloside Ⅴ	(Ju et al., 2008)
24	Astraverrucin Ⅰ	(Ju et al., 2008)
25	Astragaloside Ⅷ	(Sanchir, 1974)
26	Soyasaponin Ⅰ	(Sanchir, 1974)
27	Neoastragaloside Ⅰ	(Sellami et al., 2011)
28	Cycloastragenol	(Sellami et al., 2011)
29	Agroastragalosides	(Sellami et al., 2011)
30	SoyasapogenoⅠB	(Sellami et al., 2011)
31	Isocyclocanthosides E	(Dae-Young et al., 2013)
32	Agroastragaloside Ⅴ	(Dae-Young et al., 2013)
33	Mongholicoside A	(Dae-Young et al., 2013)
34	Mongholicoside B	(Yu et al., 2007)
35	Huangqiyenins II	(Cheng, 1994)
36	Huangqiyenins B	(Cheng, 1994)
39	Azukisaponin V	(Zhang et al., 2015)
40	Azukisaponin V methyl ester	(Zhang et al., 2015)
41	Astragaloside VIII methyl ester	(Zhang et al., 2015)
42	Robinioside F	(Yahara et al., 2000)
43	Robinioside B	(Yahara et al., 2000)

**TABLE 4:** Saponin components of astragalus membranaceus.

Compounds	R1	R2	R3	R4
Agroastragalosides	H	2′-O-AcXyl	O-Glc	OH
Agroastragalosides I	H	2, ‘3′-O-Ac2Xyl	O-Glc	OH
Astramembranosides B	H	Glc -(1–2)-Xyl	OH	H
Cyclocanthosides E	H	Xy1	O-Glc	OH
Agroastragaloside V	H	2′-O-AcXyl	O-Glc	H
Mongholicoside A	OH	Xyl	a-OH	OH
Mongholicoside B	OH	Glc	O	OH
Huangqiyenins II	H	H	O	OH
Huangqiyenins B	H	Glc	O	OH

**TABLE 5:** Saponin components of astragalus membranaceus.

Compounds	R1	R2	R3	R4
Acetylastragaloside I	3′,4′-O-Ac2Xyl	O-Glc	H	H
Agroastragalosides II	2′-O-Ac2Xyl	O-Glc	H	H
Agroastragalosides IV	3′-O-AcXyl	O-Glc	H	H
Astragaloside II	H	O-Glc	H	Glc
Astragaloside III	2′,3′,4′-O-Ac2Xyl	O-Xyl	H	H
Astragaloside IV	2′,3′-O-Ac2Xyl	O-XylO-Xyl	H	H
Astragaloside I	2′,4′-O-Ac2Xyl	O-Xyl	H	H
Astramembranosides A	3′-O-AczXyl	O-Xyl	H	H
Astramertbrannin II	2′-O-AcXyl	O-Xyl	H	H
Astraverrucin I	Rha-(1→4)-Glc	OH	H	H
Brachyosides B	Xyl	O-Glc	Ac	H
Cycloastragenol	Rha-(1→2)-(6′-O-AcXyl)	OH	H	H
Isoastragaloside II	2'-O-AcXyl	O-Rha	H	H
Isoastragaloside I	Xyl	O-Rha	H	H
Isoastragaloside IV	Ara-(1→2)-Xyl	OH	H	
Isoastragaloside V	Ara-(1→2)-Xyl	O-Xyl	H	H
Isoastragaloside VI	2',3'-O-Ac2Xyl	OH	H	H
Isoastragaloside VII	H	OH	H	H
Neoastragaloside I	2',3'-O-Ac2Xy1	O-Xyl	H	H
Huangqiyenins I	H	O	H	H
Huangqiyenins A	Glc	O	H	H

### 2.3 Polysaccharide components

**FIGURE 5:**
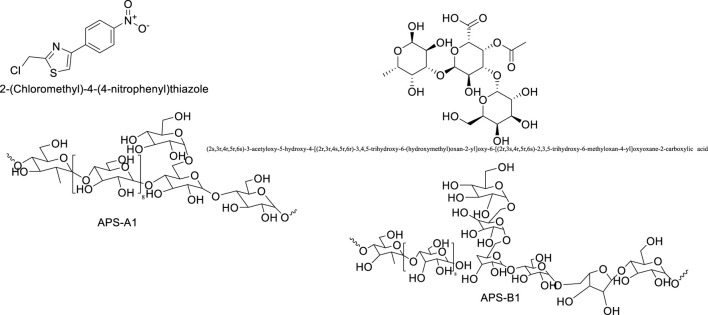
The structural formula of Astragalus membranaceus.

Astragalus polysaccharides are light yellow powders mainly derived from the dried roots and stems of Astragalus mongholicus and Astragalus membranaceus. They consist of substances such as glucose, hexuronic acid, and fructose, and have properties such as enhancing physical health, antiviral effects, and anti-fatigue. Astragalus polysaccharides can promote biochemical processes in B cells of lymph nodes and enhance the phagocytic capacity of mononuclear macrophages. In addition to protecting target cells, they can resist T cell activity, leading to the production of more immune cells and thus improving physical vitality. Experimental studies show that Astragalus membranaceus can resist infections caused by *Mycobacterium tuberculosis*, and it exhibits certain antagonistic effects against *Shigella dysenteriae*, *Streptococcus* pneumoniae, and *Staphylococcus aureus*. Polysaccharide Components of Astragalus Membranaceus are summarized in Table 6. The structural formula of Astragalus Membranaceus is shown in Figure 5.

**TABLE 6:** Polysaccharide components of astragalus membranaceus.

Serial no.	Compound name	Composition (mol)	Glycosidic bonds	Refences
1	APS-I	Arabinose, Xylose, and Glucose (2.32: 4.29: 93.39)	β-(1→4)-glycosidic bond、β-(1→6)-glycosidic bonds	Zhu et al. (2011)
2	APS-II	Arabinose, Xylose, and Glucose (0.62: 2.74: 95.97)	β-(1→4)-glycosidic bond、β-(1→6)-glycosidic bonds	Zhu et al. (2011)
3	RAP	Rhamnose, Arabinose, Glucose, Galactose, and Galacturonic Acid (0.03: 1.00:0.27: 0.36: 0.30)	β-(1→4)-glycosidic bond、β-(1→6)-glycosidic bonds	Yin et al. (2012a)
4	Polysaccharide from water	Mannose, Rhamnose, Galacturonic Acid, extract of Radix Astragali, and Glucose (1.1: 2.2: 76.5: 7.7: 4.2: 5.0)	β-(1→4)-D-glucanr、β-(1→6)-glycosidic bonds	Yin et al. (2012b)
5	APs-1-1	Rhamnose, Arabinosea, Xylose, and Glucose (1.00: 5.91: 16.24: 49.56)	β-(1→4)-glycosidic bond、β-(1→6)-glycosidic bonds	Mao et al. (2009)
6	APs-3-1	Rhamnose, Arabinose, Xylose, Glucose, (1.00: 1.82: 11.04: 23.23:22.51: 1.32: 5.20)	β-(1→4)-glycosidic bond、β-(1→6)-glycosidic bonds	Mao et al. (2009)
7	APS	Galactose, Glucuronic Acid and Galacturonic Acid, L-Rhamnose, D-Xylose, L-Xylose, D-Ribose, L-Ribose, L-Arabinose, D-Galactose, M-D-Glucose, D-Mannose (5.35: 3.49: 2.24: 2.65: 1.20: 5.85: 10.18: 15.53: 48.76)	β-(1→4)-glycosidic bond、β-(1→6)-glycosidic bonds	Mao et al. (2009)
8	RAPS	D-Ribose, D-Arabinose, L-Rhamnose, D-Mannose, D-Glucose, and D-Galactose (1.0: 14.1: 0.3: 19.9: 181.3: 6.3)	β-(1→4)-glycosidic bonds、β-(1→3)-glycosidic bonds、α-(1→6)-glycosidic bonds	Mao et al. (2009)
9	APS	Mannose, Glucose, Xylose, Arabinose, Glucuronic Acid, Rhamnose	β-(1→4)-glycosidic bond、β-(1→6)-glycosidic bonds	Zeng et al. (2018)
10	AERP1	Mannose, Rhamnose, Galacturonic Acid, Glucose, Galactose, and Arabinose (1.00: 2.59: 12.15: 2.60:3.07: 4.54)	β-(1→4)-glycosidic bond、β-(1→6)-glycosidic bonds	Zeng et al. (2018)
11	AERP2	Glucose–AX-I-3b–Arabinose, Xylose, and Glucose (10.4:79.3: 1.11)	β-(1→3)-glycosidic bonds、β-(1→6)-glycosidic bonds	Zeng et al. (2018)
12	APS4	Rhamnose, Arabinose, Xylose, Mannose, Glucose, Galactose (0.3: 0.6: 1.0: 1.0: 12.1:1.7)	β-(1→4)-glycosidic bond、β-(1→6)-glycosidic bonds	Liu et al. (2019)
13	APS90	Rhamnose, Arabinose, Xylose, Mannose, Glucose, Galactose (0.6: 0.8: 0.7: 1.0: 12.3: 2.0)	β-(1→4)-glycosidic bond、β-(1→6)-glycosidic bonds	Liu et al. (2019)
14	APS-I	Rhamnose, Galacturonic Acid, Galactose, Glu, and Arabinose (0.1: 0.39: 13.4: 17.2: 1)	β-(1→4)-glycosidic bond、β-(1→6)-glycosidic bonds	Li et al. (2019)
15	APS-II	Arabinose (10.3 0.14: 0.14: 9.6: 24.04: 1)	β-(1→4)-glycosidic bond、β-(1→6)-glycosidic bonds	Li et al. (2019)
16	APS3a	Rhamnose, Galacturonic Acid, Galactose, Arabinose, Glucose, Glucuronic Acid, and Fuc	β-(1→4)-glycosidic bond、β-(1→6)-glycosidic bonds	Prévost et al. (1987)
17	PS-II	Glucuronic Acid, Rhamnose, Galacturonic Acid, Glucose, Galactose, and Arabinose (0.32: 0.56: 1.70: 79.85: 3.86: 13.71)	β-(1→4)-glycosidic bond、β-(1→6)-glycosidic bonds	Prévost et al. (1987)
18	APOS	Glucuronic Acid, Rhamnose, Galacturonic Acid, Glucose, Galactose, and Arabinose (0.25: 0.30: 1.58: 93.70: 1.40: 2.76)	β-(1→4)-glycosidic bonds、α-(1→6)-glycosidic bonds、β-(1→3)-glycosidic bonds	Fan et al. (2024)
19	APS2-1	Mannose, Rhamnose, Glucuronic Acid, Galacturonic Acid, Glucose, Galactose, Xylose, and Arabinose (2.3: 4.8: 1.7: 14.0: 5.8: 11.7: 2.8: 12.6)	β-(1→4)-glycosidic bond、β-(1→6)-glycosidic bonds	Fan et al. (2024)
20	APS3-1	Rhamnose, Galacturonic Acid, Glucose, Galactose, and Arabinose (0.8: 2.3: 0.8: 2.3: 4.1)	β-(1→4)-glycosidic bond、β-(1→6)-glycosidic bonds	Wu et al. (2021)
21	HAPS2a	Mannose, Rhamnose, Glucuronic Acid, Galacturonic Acid, Glucose, Galactose, and Arabinose (0.22: 1.47: 0.27: 0.22: 2.44: 50.76: 44.62)	β-(1→4)-glycosidic bond、β-(1→6)-glycosidic bonds	Wu et al. (2021)
22	APS2a	Arabinose (988.9 0.90: 10.05: 1.10: 8.76: 2.05: 33.52: 43.62)	β-(1→4)-glycosidic bond、β-(1→6)-glycosidic bonds	Wu et al. (2021)
23	APS-A1	Glucose, Galactose, and Arabinose (52.3: 1.0: 1.3)	β-(1→4)-glycosidic bond、β-(1→6)-glycosidic bonds	Li et al. (2022)
24	APS-B1	Glucose, Galactose, Arabinose, Mannose, Rhamnose, and Galacturonic Acid (75.2: 17.3: 19.4: 1.0: 1.1: 1.3)	β-(1→4)-glycosidic bond、β-(1→6)-glycosidic bonds	Li et al. (2022)
25	AP1	Arabinose, Glucose, Galacturonic Acid, Glucuronic Acid	β-(1→4)-glycosidic bond、β-(1→6)-glycosidic bonds	Tian et al. (2023)
26	AP2	Arabinose, Galactose, Glucose, Galacturonic Acid, and Glucuronic Acid	β-(1→4)-glycosidic bond、β-(1→6)-glycosidic bonds	Tian et al. (2023)

### 2.4 Amino acid components

Astragalus membranaceus contains 25 amino acids, including γ-aminobutyric acid, asparagine, aspartic acid, threonine, serine, glutamic acid, proline, glycine, alanine, cysteine, methionine, isoleucine, leucine, and others. In addition, Astragalus membranaceus also contains trace elements, caffeic acid, chlorogenic acid, coumarin, nicotinic acid, riboflavin, vitamin P, starch E, sterols, folic acid, linolenic acid, linoleic acid, betaine, choline, and others. Amino acids can be synthesized into immunoglobulins in the human body, thereby playing an essential role in protecting the body and enhancing immune function. Amino acids participate in normal metabolism, which maintains nutritional balance, supports normal physiological functions, improves sleep quality, promotes growth and development, as well as regulates blood pressure.

### 2.5 Other components

Astragalus membranaceus contains various chemical components other than amino acids, such as trace elements [e.g., Scandium (Sc), Selenium (Se)], sucrose, vitamin D, mucilage, bitter substances, amylase, linoleic acid, coumarin, riboflavin, vanillic acid, nicotinic acid, iso-ferulic acid, ferulic acid, chlorogenic acid, caffeic acid, niacin, and others. Trace elements play an important role in maintaining human health, promoting growth and development, maintaining physiological functions, enhancing immunity, providing antioxidant effects, and supporting cardiovascular health.

## 3 Chemical composition of Dioscorea opposita

Dioscorea opposita, as a traditional Chinese medicinal herb and a food supplement, holds significant importance in TCM. This importance is deeply rooted in its rich chemical composition, as well as its unique pharmacological effects. Through years of research and accumulation, we have gradually recognized the various active ingredients contained in Dioscorea opposita (Kong, 2009). The components of Dioscorea opposita are summarized in Table 4, based on a review of the literature.

### 3.1 Polysaccharide components

The polysaccharides of Dioscorea opposita include homopolysaccharides, heteropolysaccharides, and glycoproteins, with the main sugar components being glucose, galactose, rhamnose, arabinose, xylose, mannose, and glucuronic acid. All of which are equipped with abilities to enhance the immune function of the body, improve resistance to pathogens, and help the body fight diseases. They possess antioxidant capabilities, which can eliminate free radicals in the body, reduce cellular oxidative damage, and thus play a role in delaying aging. They also promote insulin secretion and increase the activity of insulin-related enzymes, thereby reducing blood sugar levels. Additionally, they can enhance the phagocytic function of white blood cells, increase the body’s immunity, and inhibit the growth and spread of tumor cells. As a quality dietary fiber, they can promote intestinal peristalsis, increase stool volume, soften stool, and thus improve digestive issues such as constipation. Polysaccharide components of Dioscorea opposita are summarized in Table 7.

**TABLE 7:** Polysaccharide components of Dioscorea opposita.

Serial no.	Compound name	References
1	Starch polysaccharides (Polysaccharides composed of α-glucose molecules) Polygalactan (Oligosaccharides composed of multiple galactose molecules connected by β-1,4-glycosidic bonds.	(Tian et al., 2023)
2	Usually found in natural forms in cell walls and intercellular matrices)	(Tian et al., 2023)
3	Mucopolysaccharides (Primarily composed of glucose, galactose, and other monosaccharide molecules, linked together to form polymers through glycosidic bonds	(Tian et al., 2023)
4	Polyfructan (Primarily composed of fructose molecules, typically linked together through β-2,1-glycosidic bonds to form a polysaccharide structure)	(Tian et al., 2023)
5	Pectin	(Tian et al., 2023)
6	CYP	(Tian et al., 2023)
7	CYPN-Ⅰ	(Tian et al., 2023)
8	CYPN-Ⅱ	(Tian et al., 2023)
9	CYPA-I	(Tian et al., 2023)
10	CYPA-Ⅱ	(Tian et al., 2023)
11	CYPA-Ⅲ	(Tian et al., 2023)
12	DTTP	(Tian et al., 2023)
13	DOP-1	(Tian et al., 2023)
14	DOP-2	(Tian et al., 2023)
15	β-D-Glucan	(Tian et al., 2023)
16	DOPA-1	(Tian et al., 2023)
17	DOPA-2	(Tian et al., 2023)
18	Pectic Polysaccharides	(Tian et al., 2023)
19	Glycoprotein	(Tian et al., 2023)
20	Lipopolysaccharide	(Tian et al., 2023)
21	RDPS-I	(Tian et al., 2023)
22	DFPN-I	(Tian et al., 2023)
23	DFPA-I	(Tian et al., 2023)
24	YPa-Ⅰ	(Tian et al., 2023)
25	YPa-Ⅱ	(Tian et al., 2023)
26	YPbs	(Tian et al., 2023)
27	Mannan	(Tian et al., 2023)
28	Arabinogalactan	(Tian et al., 2023)
29	α-D-Mannopyranoside	(Tian et al., 2023)
30	1,2,3,4,6-Penta-O-acetyl-α-D-mannopyranose	(Tian et al., 2023)
31	1,3,4,6-Tetra-O-acetyl-β-D-mannopyranose	(Tian et al., 2023)
32	2,3,4,6-Tetra-O-acetyl-β-D-mannopyranosyl Azide	(Tian et al., 2023)
33	2,3,4,6-Tetra-O-acetyl-α-D-mannopyranosyl fluoride	(Tian et al., 2023)
34	Fluoride	(Tian et al., 2023)
35	α-D-Mannopyranosyl Fluoride	(Tian et al., 2023)
36	1,2,3,4,6-Penta-O-benzoyl-α-D-mannopyranose	(Tian et al., 2023)
37	Phenyl2,3,4,6-Tetra-O-acetyl-1-thio-α-D-mannopyranoside	(Tian et al., 2023)
38	2-O-Benzyl-1,3,4,6-tetra-O-acetyl-α-D-mannopyranose	(Tian et al., 2023)
39	1,2,3,4,6-Penta-O-acetyl-β-D-mannopyranose	(Tian et al., 2023)

### 3.2 Saponin components

Dioscorea opposita saponins are a class of natural active ingredients extracted from the Dioscorea opposita of the Dioscoreaceae family, which belongs to the steroidal saponin class of compounds. Its structure is composed of hydrophobic steroidal saponins (such as diosgenin) and hydrophilic sugar chains, which are amphiphilic and easy to form foam, so they are called “saponins”. Saponin components of Dioscorea Opposita are summarized in Table 8.

**TABLE 8:** Saponins components of Dioscorea opposita.

Serial no.	Compound name	Refences
1	Dioscin	Lenssen et al. (1994)
2	Dioscin I	Lenssen et al. (1994)
3	Diosgenin	Lenssen et al. (1994)
4	Sesquiterpene oligoglycoside	Lenssen et al. (1994)
5	Artemisinina	Lenssen et al. (1994)
6	Saponin H	Chen (2011)
7	Dioscoreside C	Chen (2011)
8	Protodioscin	Chen (2011)
9	Pseudoprotodioscin	Chen (2011)
10	Methylprotodioscin	Chen (2011)
11	Gracillin	Chen (2011)
12	Dioscoresides A	Chen (2011)
13	Dioscoresides B	Chen (2011)
14	Dioscorea saponin A	Chen (2011)
15	Dioscorea saponin B	Chen (2011)
16	Gracillin	Li et al. (2015)
17	Tokoronin	Li et al. (2015)
18	Oleanolic acid saponins	Li et al. (2015)
19	Hederagenin derivatives	Li et al. (2015)
20	Batatasin I	Ju et al. (2014)
21	Batatasin II	Ju et al. (2014)
22	Batatasin III	Ju et al. (2014)
23	Batatasin IV	Ju et al. (2014)
24	Batatasin V	Ju et al. (2014)
25	Dioscin-3-O-β-D-glucopyranoside	Ju et al. (2014)
26	Dioscorea oppositaogenin	Ju et al. (2014)
27	Dioscorea saponin A	Ju et al. (2014)
28	Dioscorea saponin B	Ju et al. (2014)
29	Dioscorea saponin C	Ju et al. (2014)
30	Oleanolic acid 3-O-β-D-glucuronide	Ju et al. (2014)
31	Oleanolic acid 28-O-β-D-glucoside	Yang et al., 2009b
32	Hederagenin 3-O-α-L-arabinoside	Yang et al., 2009b
33	Hederagenin 28-O-β-D-glucoside	Yang et al., 2009b
34	Betulinic acid 3-O-β-D-glucoside	Yang et al., 2009b
35	Dammarenediol saponins	Ma et al. (2005)
36	Quercetin	Ma et al. (2005)
37	Kaempferol	Ma et al. (2005)

### 3.3 Polyphenol components

The polyphenols of Dioscorea opposita are a class of natural plant polyphenolic compounds extracted from Dioscorea opposita, mainly including phenolic acids (such as chlorogenic acid and ferulic acid), flavonoids (such as rutin and quercetin), as well as other condensed tannins. Polyphenols are one of the important active components of Dioscorea opposita and have significant antioxidant and bioactive properties. Polyphenol components of Dioscorea Opposita are summarized in Table 9.

**TABLE 9:** Polyphenols components of Dioscorea opposita.

Serial no.	Compound name	References
1	Quercetin-3-O-glucoside	Nagai et al. (2006)
2	Rutin	Nagai et al. (2006)
3	Kaempferol-3-O-galactoside	Nagai et al. (2006)
4	Apigenin	An et al. (2015)
5	Luteolin	An et al. (2015)
6	Apigenin-7-O-glucoside	Nagai et al. (2006)
7	Luteolin-4′-O-glucoside	Nagai et al. (2006)
8	Chrysin	Zhang and Hu (2010)
9	Chlorogenic Acid	Zhang and Hu (2010)
10	Caffeic Acid	Zhang and Hu (2010)
11	Ferulic Acid	Zhang and Hu (2010)
12	Gallic Acid	Zhang and Hu (2010)
13	Condensed Tannins	Zhang and Hu (2010)
14	Catechins	Zhang and Hu (2010)
15	Anthocyanins	Zhang and Hu (2010)
16	Mucoprotein	Zhang and Hu (2010)
17	Glycoprotein	Zhang and Hu (2010)

### 3.4 Other components

**FIGURE 6:**
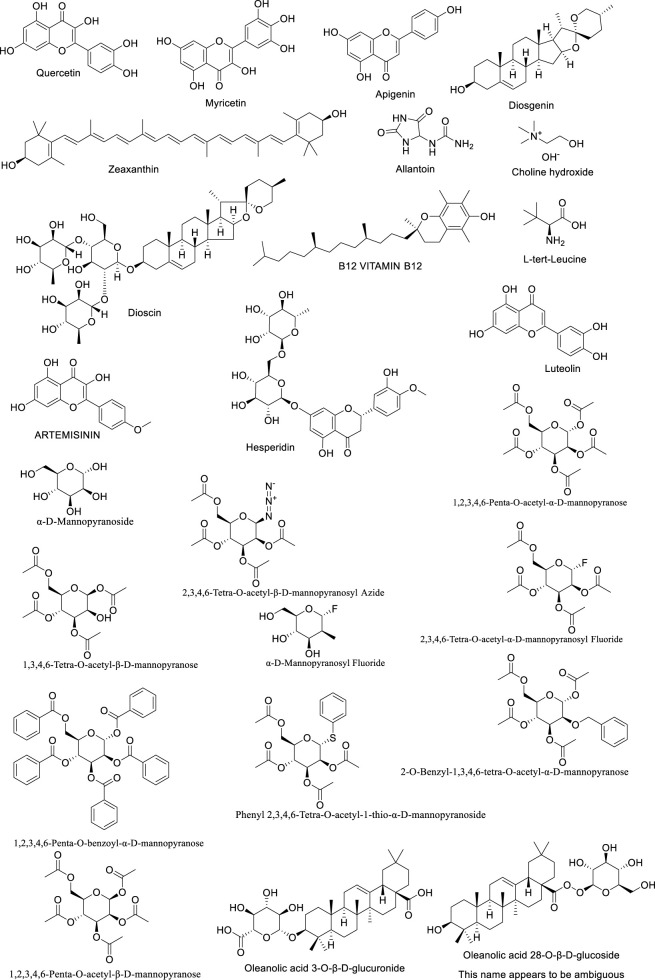
The structural formula of Dioscorea opposita.

**FIGURE 7:**
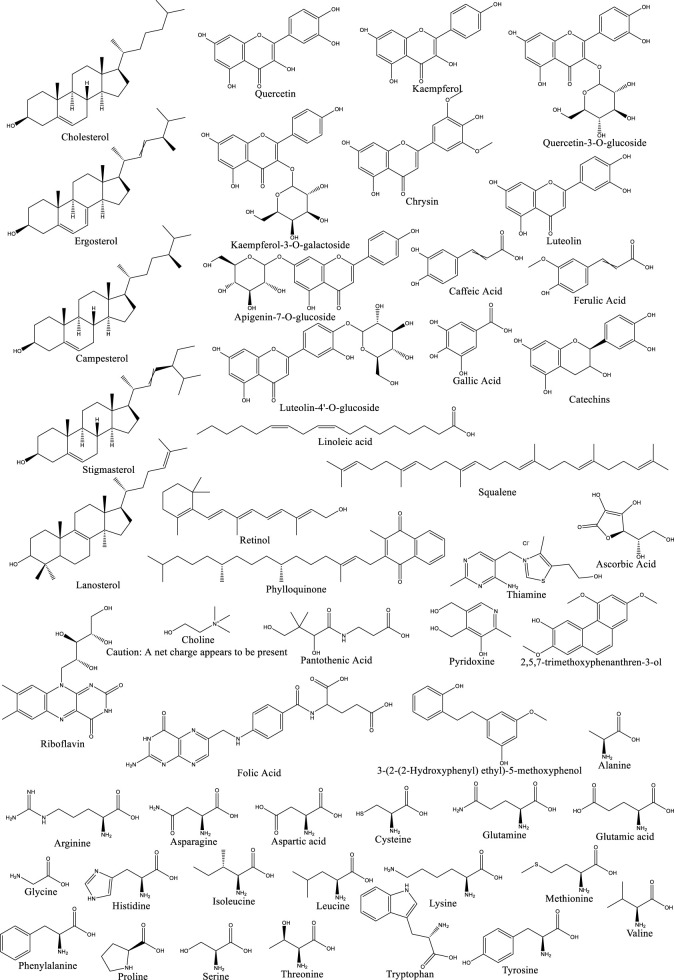
The structural formula of Dioscorea opposita.

**FIGURE 8:**
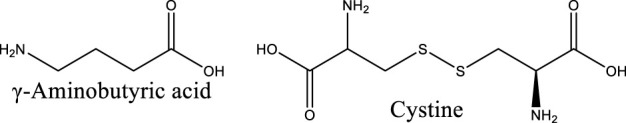
The structural formula of Dioscorea opposita.

In addition to saponins and polyphenols, Dioscorea opposita is rich in active components such as polysaccharides, allantoin, amino acids, trace elements (such as zinc and selenium), and dietary fiber. Among these, Dioscorea opposita polysaccharides are the core functional substances that possess immune regulation, anti-tumor, and intestinal probiotic effects, which can improve health by activating macrophages and promoting the production of short-chain fatty acids. Allantoin promotes the repair of epidermal cells and is widely used in skin repair products. Amino acids and trace elements work synergistically to enhance antioxidant capacity and delay aging, while dietary fiber helps regulate blood sugar, blood lipids, and improve intestinal peristalsis. These components have applications in food, medicine, and cosmetics, such as the development of functional beverages, wound dressings, and anti-aging skincare products, reflecting the multidimensional value of Dioscorea opposita as “food and medicine from the same source.” Other Components of Dioscorea Opposita are summarized in Table 10. The structural formula of Dioscorea Opposita is shown in Figures 6–8.

**TABLE 10:** Other components of Dioscorea opposita.

Serial no.	Compound name	Reference
1	Amylase	Fang et al. (2009)
2	Polyphenoloxidase	Fang et al. (2009)
3	Linoleic acid	Fang et al. (2009)
4	Linolenic acid	Fang et al. (2009)
5	Cholesterol	Fang et al. (2009)
6	Ergosterol	Fang et al. (2009)
7	Campesterol	Fang et al. (2009)
8	Stigmasterol	Fang et al. (2009)
9	β-Sitosterol	Ma et al. (2017b)
10	Squalene	Chen et al. (2019)
11	Lanosterol	Chen et al. (2019)
12	Penta sterol	Chen et al. (2019)
13	2,5-D-Spirostane-3,5-dione	Chen et al. (2019)
14	Retinol	Chen et al. (2019)
15	Ascorbic Acid	Chen et al. (2019)
16	Tocopherol	Chen et al. (2019)
17	Phylloquinone	Chen et al. (2019)
18	Thiamine	Chen et al. (2019)
19	Riboflavin	Tsukui et al. (1999)
20	Niacin	Tsukui et al. (1999)
21	Choline	Tsukui et al. (1999)
22	Pantothenic Acid	Tsukui et al. (1999)
23	Pyridoxine	Tsukui et al. (1999)
24	Folic Acid	Tsukui et al. (1999)
25	2,5,7-trimethoxyphenanthren-3-ol	Tsukui et al. (1999)
26	1-(3-Hydroxy-5-methoxyphenyl)-2	Tsukui et al. (1999)
27	1-(3-hydroxyphenyl) ethane4	Yakuwa et al. (1981)
28	3-(2-(2-Hydroxyphenyl) ethyl)-5-methoxyphenol	Yakuwa et al. (1981)
29	Alanine	Yakuwa et al. (1981)
30	Arginine	Yakuwa et al. (1981)
31	Asparagine	Yakuwa et al. (1981)
32	Aspartic acid	Yakuwa et al. (1981)
33	Cysteine	Yakuwa et al. (1981)
34	Glutamine	Yakuwa et al. (1981)
35	Glutamic acid	Chen et al. (2011)
36	Glycine	Chen et al. (2011)
37	Histidine	Chen et al. (2011)
38	Isoleucine	Chen et al. (2011)
39	Leucine	Mitsunaga et al. (2004)
40	Lysine	Mitsunaga et al. (2004)
41	Methionine	Mitsunaga et al. (2004)
42	Phenylalanine	Bai et al. (2008)
43	Proline	Bai et al. (2008)
44	Serine	Bai et al. (2008)
45	Threonine	Bai et al. (2008)
46	Tryptophan	Bai et al. (2008)
47	Tyrosine	Bai et al. (2008)
48	Valine	Bai et al. (2008)
49	Cystine	Bai et al. (2008)
50	γ-Aminobutyric acid	Bai et al. (2008)

## 4 Pharmacological effects

### 4.1 Inhibition of tumor angiogenesis

**FIGURE 9:**
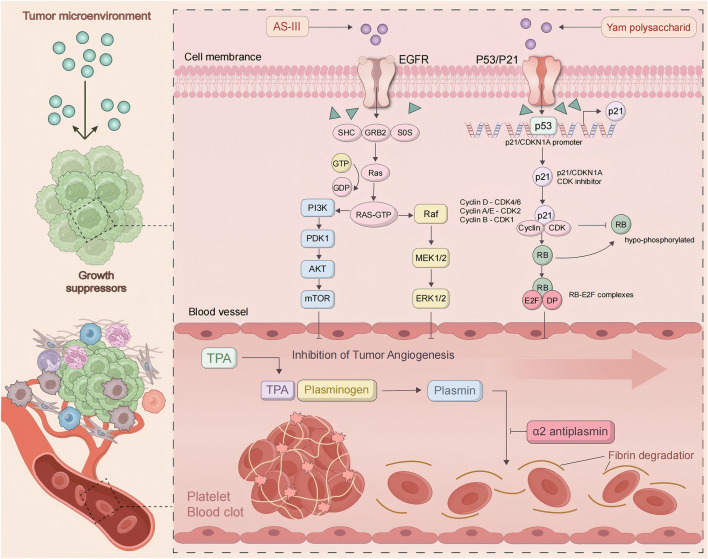
Mechanism of anti-cancer effects of Astragalus membranaceus-Dioscorea opposita in inhibiting tumor angiogenesis.

The generation of blood vessels within tumors is a key step in tumor growth and metastasis. As is well known, tumors are nourished by nutrients and oxygen via angiogenesis (Bergers and Benjamin, 2003). Moreover, the newly formed blood vessels in tumors provide the structure and pathways for tumor expansion and metastasis, increasing the likelihood of spreading and infecting other tissues. Vascular endothelial growth factor (VEGF) plays a crucial role in promoting tumor angiogenesis, while other cytokines and signaling pathways may function importantly in inhibiting tumor blood vessel formation. By observing the effective anti-cancer action of Astragalus membranaceus and Dioscorea opposita, and then the individual and combined effects of Astragalus membranaceus-Dioscorea opposita on tumor angiogenesis *in vitro*, as well as their effects on the EGFR/PI3K/AKT and HIF-1α/VEGF signaling pathways, further elucidate the anti-cancer mechanisms of Astragalus membranaceus-Dioscorea opposita in clinical practice and the intrinsic material basis of commonly used medicinal combinations (Cao, 2023; He, 2016). Experimental studies by Gao Liufang et al. found that Astragalus-Dioscorea powder protects against acute alcohol-induced gastric mucosal injury in rats (Gao et al., 2022). Cao Li et al. explored the effects of Dioscorea extract on the growth of Chinese hamster ovary (CHO) cells and found that at a concentration of 5 mg/mL, Dioscorea extract significantly promoted the cell vitality and growth of CHO cells, at least partly due to increased SOD activity and the reduced MDA content (Cao, 2023). Furthermore, *in vitro* models (Li et al., 2015; Cao, 2023; Xiang et al., 2013) show that Astragalus-Dioscorea polysaccharides exert anti-proliferative and antioxidant activity towards cancer cell lines. They inhibit the protein expressions of aging-related genes such as P53 and P21, resist oxidative stress, and regulate the activity of antioxidant enzymes in the body. Furthermore, they degrade enzyme precursors and promote the activation of tumor cell apoptosis proteins such as caspase-3 and caspase-8. Astragalus-Dioscorea polysaccharides can control tumor cell growth, decrease scratch healing rate, and promote tumor cell apoptosis. In SGC-7901 cells, treatment with AS-IV (Yang et al., 2021) reduced the activity of cyclooxygenase-2 (COX-2), cut the production of prostaglandin E2, and downregulated VEGF expression, ultimately inhibiting tumor growth. In studies on A549 and U251 cells, it was also found that AS-IV reduced VEGF expression. In endothelial cells, AS-III induces embolization through catheter-directed arterial chemotherapy, activates the epidermal growth factor receptor (EGFR), and subsequently the phosphorylation of AKT, ERK1/2, and growth factor signaling pathways (Fang et al., 2024). In mice with H22 xenograft tumors, the protein expressions of VEGF, MMP-2, MMP-9, aquaporin-1, and platelet-endothelial cell adhesion molecule-1 (CD31) were decreased after AS-IV treatment. These results indicate that AS-IV inhibits H22 ascites-associated hepatocellular carcinoma (HCC) development by suppressing angiogenesis, cell migration, and fluid transportation. In H1299 cells, lupenol strongly attracted VEGFR2 and inhibited the production of chemotactic factors, thereby blocking the progression of lung cancer (Li et al., 2013). These studies suggest that the combination of Astragalus membranaceus and Dioscorea opposita in cancer treatment can improve the body’s immune function and quality of life, accelerate tumor regression, and increase the complete regression rate. Astragalus can improve the tumor microenvironment by regulating amino acid metabolism and antioxidant stress (eliminating free radicals), reducing the release of inflammatory factors, and indirectly inhibiting tumor progression. Dioscorea opposita polysaccharides and saponins can induce apoptosis in tumor cells (such as breast cancer and melanoma) and block tumor cell migration and invasion by inhibiting the expression of matrix metalloproteinases (MMP-2/MMP-9), reducing the risk of metastasis. Together, they optimize the tumor microenvironment, reduce inflammation and oxidative damage, creating more favorable conditions for immunotherapy and chemotherapy. These pathways represent critical targets in current cancer therapy. For instance, VEGF inhibitors have been applied in clinical practice, and their efficacy has been validated in various cell lines (such as A549 and U251) and animal models (including H22). This aligns well with clinically established anti-tumor mechanisms, particularly anti-angiogenesis, thereby providing a theoretical basis for clinical translation. However, *in vitro* models cannot fully replicate the complex tumor microenvironment in humans, and there exist differences in pathophysiological characteristics between animal models and human tumors. Consequently, the reliability of translating these preclinical results to clinical settings requires further verification. Mechanism of Anti-Cancer Effects of Astragalus membranaceus-Dioscorea opposite in Inhibiting Tumor Angiogenesis is shown in Figure 9.

### 4.2 Inhibition of tumor cell proliferation

**FIGURE 10:**
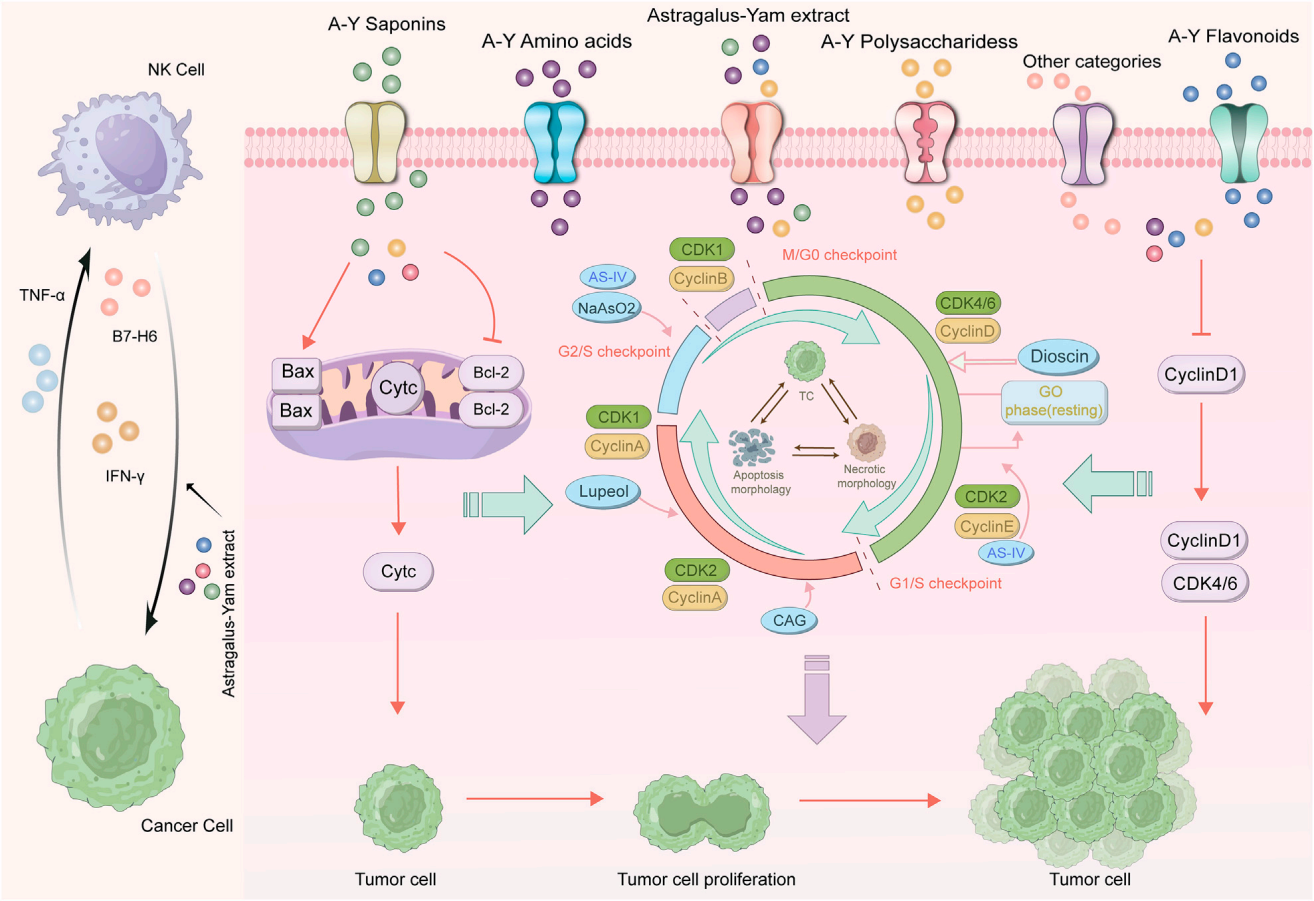
Mechanism of anti-cancer effects of Astragalus membranaceus-Dioscorea opposita in suppressing tumor cell cycle dysregulation.

The continuous proliferation of tumor cells is a key indicator of malignant tumors. By regulating relevant signaling pathways and the cell cycle, Astragalus membranaceus-Dioscorea opposita can suppress the proliferation of lung cancer, colon cancer, liver cancer, colorectal cancer (CRC), and vulvar squamous cell carcinoma (VSCC). The process by which Astragalus membranaceus-Dioscorea opposita regulates the cell cycle and pathways to inhibit tumor cell proliferation is as follows: In CRC cells, the expression of cyclin D1 and CDK4 is inhibited by AS-IV, causing cell cycle arrest at the G0/G1 phase. In addition, tumor suppressor genes can also block the cell cycle. Zhao et al. demonstrated that AS-IV upregulates the expression of type II transforming growth factor-beta receptor (TGF-βRII) and SMAD4, thereby reversing the inhibition of the TGF-β/Smad signaling pathway and ultimately causing cell cycle arrest at the G0/G1 phase (Zhao et al., 2019). This mechanism provides insights into adjuvant therapy for colorectal cancer after surgery in clinical practice. Multiple clinical retrospective studies have demonstrated that traditional Chinese medicine formulas containing Astragalus membranaceus (e.g., Huangqi Jianzhong Decoction) combined with the FOLFOX chemotherapy regimen can reduce the 2-year recurrence rate of stage III colorectal cancer patients by approximately 15% and alleviate chemotherapy-induced leukopenia (Chen et al., 2014). Furthermore, Guo et al. reported that the combination of NaAsO2 and AS-IV downregulates the expression of phosphorylated phosphoinositide 3-kinase (p-PI3K), phosphorylated protein kinase B (p-Akt), phosphorylated mammalian target of rapamycin (p-mTOR), PI3K, Akt, and mTOR, as well as other key genes in HepG2 cells, and upregulates the mRNA of G protein subunit gamma-transducin 1 (GNGT1), thereby inducing cell cycle arrest at the S phase (Guo et al., 2021). In clinical practice, a similar rationale to this combination strategy has been applied in the adjuvant regimen for interventional therapy of hepatocellular carcinoma (HCC). Specifically, following transcatheter arterial chemoembolization (TACE), the administration of *Astragalus membranaceus* extract (containing astragaloside IV, AS-IV) in combination with sorafenib has been demonstrated to prolong the median progression-free survival (mPFS) of patients with advanced HCC by 2.3 months. Additionally, this combined regimen significantly accelerates the rate of decline in serum alpha-fetoprotein (AFP) levels (Cao et al., 2016). Sun et al. revealed that AS-IV induces cell cycle arrest at the G0 phase and increases the expression of p21 both *in vitro* and *in vivo* (Bergers and Benjamin, 2003). Lupenol independently activates CDK inhibitor 2A in Hep-2 and UPCI-SCC-131 cells, but does not activate p21, thereby inhibiting the expression of cyclin D1 and ultimately causing tumor cell cycle arrest at the G2 phase (Yang et al., 2021). CAG negatively regulates the Janus kinase 2/signal transducer and activator of transcription 3 (STAT3) axis, leading to the accumulation of gastric cancer (GC) in the G1 phase (Choi et al., 2010). Extracellular signals are imported into the cell nucleus through specific pathways, with mitogen-activated protein kinase (MAPK) acting as a mediator to transmit the stimulus from the cell surface to the nucleus. Upon stimulation, MAPK/extracellular signal-regulated kinase (ERK) is phosphorylated and becomes active. Once it enters the nucleus, the activated ERK attaches to target genes to control physiological processes, including cell division and proliferation. Notably, AS-IV inhibits glioma growth *in vivo* and suppresses glioma cell proliferation by inhibiting the activity of the MAPK/ERK signaling pathway in U251 cells (Zhao et al., 2019). Additionally, PI3K transmits mitotic signals to target genes via Akt/mTOR to promote cell proliferation. AS-IV inhibits the course and thereby suppresses the proliferation of lung cancer and liver cancer cells (Guo et al., 2021). Moreover, lupenol treatment ot only suppresses skin cancer by inhibiting PI3K, Akt phosphorylation, nuclear factor kappa-B (NF-κB)/p65 translocation, and IKKα activation, but also antagonizes the proliferation of ocular cancer cells and tumor development *in vivo* (Bhattacharyya et al., 2017; Jiang et al., 2017). Xiang et al. used various concentrations of APS (12.5, 25.0, 50.0, 100.0 μg/mL) as the blank control group, along with dimethyl sulfoxide (DMSO) and cisplatin (2 μg/mL), and applied the MTT assay to verify the inhibitory effects of APS on the proliferation of nasopharyngeal carcinoma CNE-2 cells. Fang Zhike et al. analyzed the intervention effects of Dioscorea extract on aging rats with chronic atrophic gastritis based on the Shh pathway and found that Dioscorea extract regulated the secretion of gastric juice, gastric acid, and pepsin, while inhibiting the progression of the disease (Fang et al., 2024). In a rat model of Parkinson’s disease induced by lipopolysaccharide (LPS), treatment with dioscin was shown to improve the motor defects in Parkinson’s mice, at least partly by inhibiting the TLR/NF-κB signaling pathway (Li et al., 2017). In a co-cultured system containing 3T3-L1 adipocytes and RAW 264 macrophages, Dioscin administration was shown to suppress inflammation via down-regulating the expressions of tumor necrosis factor (TNF), nitric oxide (NO), and monocyte chemoattractant protein-1 (MCP-1), as well as the phosphorylation of c-Jun N-terminal kinase (JNK) in macrophages (Hirai et al., 2010). In a collagen-induced arthritis (CIA) mouse model, the therapeutic effects of Dioscorea saponin tablets on rheumatoid arthritis (RA) were studied, focusing on the modulation of the 5-LO/LTB4 signaling pathway (Li et al., 2018). Dioscorea saponin tablets reduced the expression of the LTB4 synthesis enzymes 5-LO and LTA4H, as well as the expression of the LTB4 receptors BLT1 and BLT2. Body weight, joint index, joint swelling, thymus index, spleen index, and serum LTB4 levels in CIA mice were all decreased. There is a complementarity between Astragalus and Dioscorea opposita in inhibiting tumor proliferation and inducing apoptosis. Astragalus primarily focuses on cell cycle arrest and signaling pathway regulation, while Dioscorea opposita enhances the execution of apoptosis and inhibits metastasis, thus jointly enhancing the anti-tumor effect. Astragalus saponins and polysaccharides exert direct anti-tumor effects by blocking the tumor cell cycle (such as G0/G1 phase), inhibiting proliferation-related signaling pathways (such as PI3K/AKT/mTOR), and inducing tumor cell apoptosis (by upregulating pro-apoptotic proteins such as Bax and Caspase-3). In clinical practice, the combined application of the two is more common: for example, when patients with advanced breast cancer receive paclitaxel chemotherapy, the addition of the Astragalus membranaceus-Dioscorea opposita compound (30 g of Astragalus membranaceus +20 g of Dioscorea opposita per day) can increase the chemotherapy response rate from 52% to 68% and reduce the incidence of neutropenia by 25%. Dioscorea opposita polysaccharides and saponin components can induce apoptosis in tumor cells (such as breast cancer and melanoma) and inhibit the expression of matrix metalloproteinases (MMP-2/MMP-9), thereby blocking tumor cell migration and invasion, which reduces the risk of metastasis. Inhibition of tumor cell proliferation is shown in Figure 10.

### 4.3 In terms of apoptosis in tumor cells

The regulation of cell death is achieved through gene control, and tumor cells achieve unlimited proliferation by reducing apoptosis. The induction of apoptosis by Astragalus membranaceus-Dioscorea opposita is crucial in the fight against tumors. Apoptosis has both endogenous and exogenous pathways: mitochondrial apoptosis and death receptor pathways. Cytochrome C (Cyt C), the caspase family, and the Bcl-2 protein family are important components of the mitochondrial apoptosis endogenous pathway. In experiments with HT29, SW480, and BALB/c nu/nu mice, AS-IV promotes tumor cell death by enhancing Cyt C, Omi, and p21 expression. AST also promotes NAG-1, Egr-1, and PARP cleavage and inhibits the PI3K/Akt signaling (Yang et al., 2021; Fang et al., 2024; Huang et al., 2021; Fosslien, 2001; Wang et al., 2020). Further research showed that Dioscorea saponins (DS) promote PARP and caspase-3 while inhibiting EGFR and STAT3, leading to apoptosis via autophagy (Tamilselvam et al., 2019). The exogenous death receptor pathway is another important route for apoptosis. AS-IV also induces apoptosis by increasing the expression of cleaved caspase-8, cleaved caspase-3, cleaved PARP, Fas, and FasL (Uemura et al., 2010; Tongzhou et al., 2017). *In vitro* cell experiments have found that Dioscorea opposita contains various polysaccharide components with antioxidant activity (Chun et al., 2020). When the concentration of Dioscorea polysaccharides is between 0.025 and 0.25 g/L, it significantly inhibits hypoxic apoptosis in nerve cells (Ju et al., 2014). Increasing the dosage of Dioscorea polysaccharides also enhances the degradation of zymogens, promotes the activation of tumor cell apoptosis-related proteins such as caspase-3 and caspase-8, weakens the cell scratch healing rate, and controls tumor cell growth (Xiang et al., 2013). Currently, studies on the relationship between Astragalus membranaceus, Dioscorea opposita, and tumor cell apoptosis are mostly concentrated on *in vitro* cell experiments and animal experiments, and have not yet been translated into mature clinical treatment regimens.

### 4.4 In terms of autophagy in tumor cells

Autophagy refers to the process by which cells transport damaged, denatured, or aging proteins and organelles to lysosomes for digestion and degradation. Under normal circumstances, this process is beneficial for maintaining cellular homeostasis. In the event of stress, autophagy can prevent the accumulation of toxic or carcinogenic damaged proteins, thereby exerting an inhibitory effect on cellular carcinogenesis. However, once cancer cells have formed, autophagy can also provide cancer cells with richer nutrients, promoting tumor growth. Modern pharmacological experiments have confirmed that Astragalus membranaceus-Dioscorea opposita has excellent anti-cancer and cancer-preventive effects. In rat models injected with the extract, the incidence of cancer was 16.28%, compared to 51.52% in the control group. In a rat model of lung cancer introduced by 3-methylcholanthrene iodized oil solution, the injection with extracts from Astragalus membranaceus-Dioscorea opposita significantly reduced tumor formation in the experimental animals. In addition, in experimental mice injected with polysaccharides, the survival period was extended from 15.71 days to 21.57 days. This effect was also observed in animals with spontaneously occurring melanomas, indicating that Astragalus membranaceus-Dioscorea opposita can extend the survival period of tumor-bearing mice. Clinical experiments in patients with cancerous ascites caused by gastric cancer and ovarian cancer also confirmed the beneficial action of Astragalus-Dioscorea polysaccharides on activating lymphocytes in cancerous ascites patients. After activation, the lymphocytes encircle the tumor cells, ultimately promoting the apoptosis of tumor cells. Astragalus membranaceus-Dioscorea opposita also has a significant reparative effect on T-cell E receptors damaged by pancreatic enzymes, restoring detached E receptors (Li et al., 2015; Yan et al., 2008). Long-term consumption improves hearing and vision, lightens the body, and prevents hunger and worry. Dioscorea opposita is often combined with Astragalus membranaceus for the purpose of strengthening the body’s resistance and consolidating the constitution in traditional Chinese medicine practice. For instance, the Astragalus-Dioscorea opposita combination in the “Nitang Qingdan Formula” regulates the AMPK metabolic pathway to promote lipid metabolism and autophagy, thereby improving muscle atrophy in patients with cancer cachexia. In the adjuvant therapy of breast cancer, Dioscorea opposita, when combined with other Chinese herbal medicines, can enhance chemosensitivity by modulating the expression of autophagy-related proteins.

**FIGURE 11:**
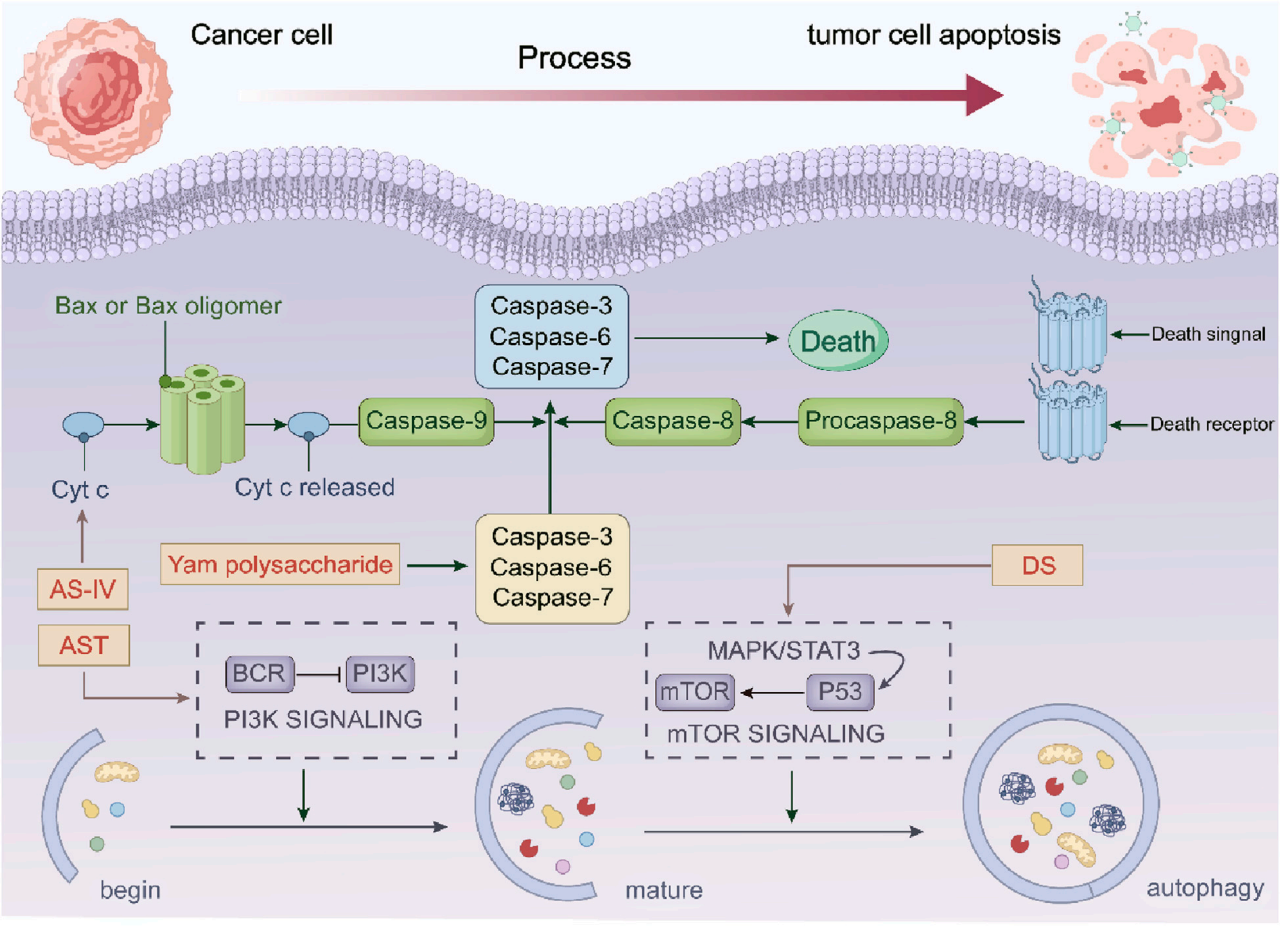
The anti-cancer mechanism of Astragalus membranaceus-Dioscorea opposita in tumor cell apoptosis and autophagy.

Future efforts should focus on strengthening basic research and clinical translation to clarify the appropriate patient populations and optimal compatibility schemes, so as to promote the precise application of this herbal combination in cancer treatment. In terms of Autophagy in tumor cells are as shown in Figure 11.

### 4.5 In terms of pyroptosis in tumor cells

The combined use of Astragalus membranaceus and Dioscorea opposita exhibits multidimensional synergistic effects in regulating cellular pyroptosis, and the underlying mechanism involves the precise regulation of inflammatory signaling pathways, immune microenvironment, and core pyroptotic molecules by their active components (Kim et al., 2010). Astragalus polysaccharides (APS), the active component derived from Astragalus membranaceus, can activate the JAK-STAT pathway to promote the secretion of interferons (IFNs) such as IFN-β and IFN-γ. These cytokines not only enhance the recognition and cytotoxicity of natural killer (NK) cells against tumor cells but also can further upregulate the expression of the pyroptosis-related protein gasdermin D (GSDMD), thereby laying a foundation for the initiation of immunogenic pyroptosis (Araki et al., 1981; Chen et al., 2024; Yang et al., 2003; Liu and Fu, 2006; Su et al., 2017; Li, 2021). Meanwhile, total flavonoids from Astragalus membranaceus (AFs) can enhance the phagocytic function of macrophages, promote the adhesion between neutrophils and endothelial cells, accelerate the clearance of inflammatory debris released during pyroptosis, and thereby prevent excessive inflammatory injury (Xu and Zhang, 2013). Dioscorea opposita polysaccharides (DOP), the active component from Dioscorea opposita, participate in the pyroptotic cascade reaction by regulating the p38 MAPK pathway. Specifically, DOP can synergize with components of Astragalus membranaceus to inhibit the abnormal activation of the NLRP3 inflammasome, reduce the cleavage and activation of caspase-1, and thereby balance the secretion rhythm of pro-inflammatory cytokines such as IL-1β and IL-18 (Shen, et al., 2023; Li et al., 2020). The inhibitory effect of the aqueous extract of their compound formula (i.e., the compound of Astragalus membranaceus and Dioscorea opposita) on sialidase can reduce the sialic acid modification level on the surface of tumor cells, enhance the recognition of tumor cells by antigen-presenting cells (APCs), and thereby indirectly promote the initiation of pyroptosis-related immune responses (Ma et al., 2017a; Yang et al., 2003). From the perspective of Traditional Chinese Medicine (TCM) theory, the efficacy of “tonifying zang-fu organs and regulating Yin and Yang” is highly consistent with the mechanism of regulating the pyroptosis threshold of Astragalus membranaceus and Dioscorea opposita revealed in modern studies. The Astragalus membranaceus and Dioscorea opposita compound not only induces immunogenic pyroptosis in tumor cells through multi-targeted effects but also maintains inflammatory homeostasis by regulating the maturation of dendritic cells (DCs) and lymphocyte activity, thereby preventing the damage to normal tissues caused by “internal accumulation of “fire-toxin”” — a TCM term referring to excessive internal inflammation. This bidirectional regulatory effect of “promoting pyroptosis” and “controlling inflammation” provides a unique advantage for its application in tumor prevention and treatment with both efficacy and safety. Future studies are needed to further elucidate its direct regulatory effect on novel pyroptotic molecules such as gasdermin E.

### 4.6 In terms of ferroptosis in tumor cells

**FIGURE 12:**
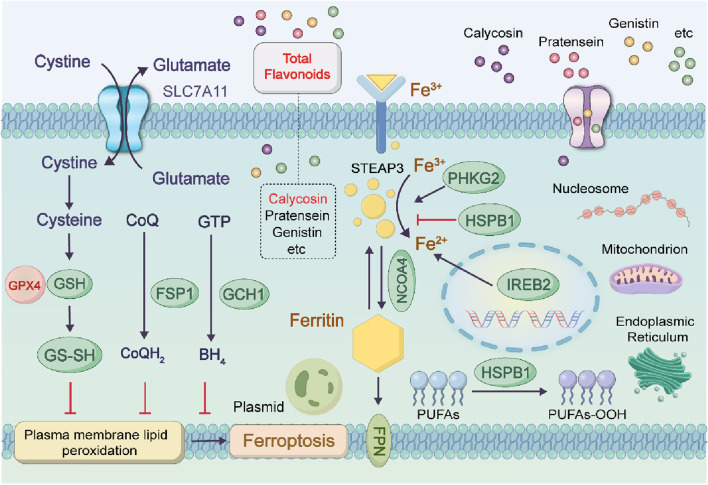
Anti-cancer mechanisms of Astragalus membranaceus-Dioscorea opposita in ferroptosis.

Ferroptosis is a form of iron-dependent regulated cell death driven by lipid peroxidation, which is closely associated with the progression of various malignant tumors and therapeutic responses. The total flavonoids in astragalus exert a neuroprotective effect on dopaminergic neurons by inhibiting ferroptosis through the modulation of the SLC7A11/GPX-4 signaling pathway. Ferroptosis is characterized by the accumulation of lipid peroxides in an iron-dependent mode. The total flavonoid components in Astragalus membranaceus exert neuroprotective effects on dopaminergic neurons by modulating the SLC7A11/GPX-4 signaling pathway and inhibiting ferroptosis (Gao et al., 2024). The combined administration of Astragalus membranaceus and Dioscorea opposita exhibits unique advantages in tumor therapy through the precise regulation of the ferroptosis mechanism, and their synergistic effect permeates the entire process of ferroptosis initiation, execution in tumor cells, and tumor microenvironment regulation (Kai et al., 2010). Furthermore, these two herbs (i.e., Astragalus membranaceus and Dioscorea opposita) can reverse ferroptosis resistance in tumor cells: Specifically, total flavonoids from Astragalus membranaceus downregulate the epithelial-mesenchymal transition-related protein Snail to restore the sensitivity of tumor cells to ferroptosis; in contrast, Dioscorea opposita polysaccharides inhibit the hypoxia-inducible factor-1α (HIF-1α)/vascular endothelial growth factor (VEGF) pathway, improve the hypoxic state of the tumor microenvironment, and thereby attenuate its protective effect against ferroptosis. This multi-target synergistic mechanism provides a novel strategy for overcoming tumor drug resistance, and its clinical translation potential warrants in-depth exploration. Although certain components of Astragalus membranaceus and Dioscorea opposita have demonstrated ferroptosis-inducing potential in in vitro experiments, their clinical translation still requires high-quality randomized controlled trials and mechanistic validation. These are shown in Figure 12.

### 4.7 In terms of cuproptosis and disulfide cell death in tumor cells

**FIGURE 13:**
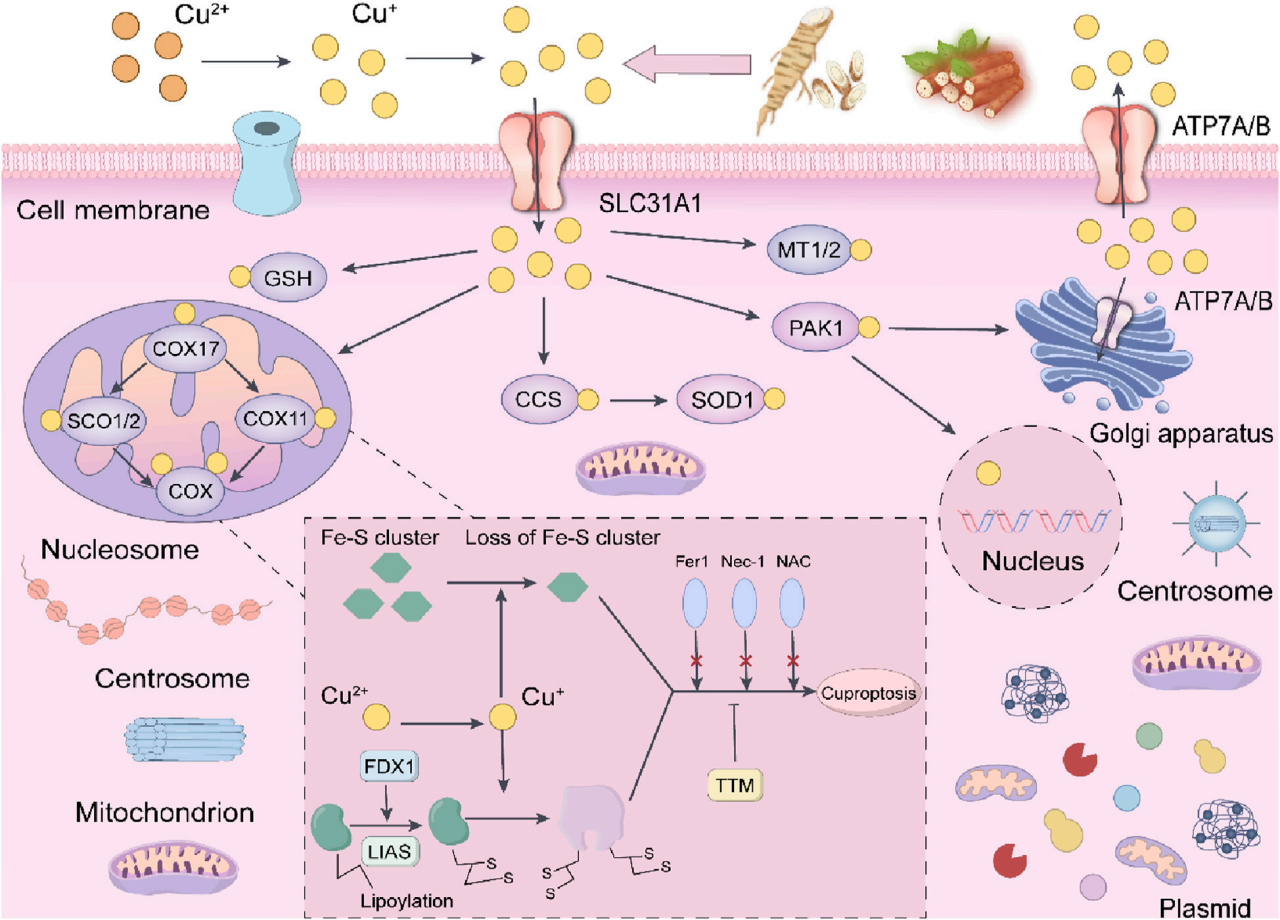
The potential mechanisms of the anti-cancer effects of Astragalus and Dioscorea opposita in relation to copper-induced death.

Autophagy, cellular necroptosis, and ferroptosis show distinct interactions and regulatory mechanisms within cells. Autophagy can influence the health status of cells by clearing damaged organelles and proteins, thus indirectly affecting the occurrence and development of necroptosis and ferroptosis. For instance, defects in autophagy may lead to increased sensitivity of cells to ferroptosis. Moreover, ferroptosis and necroptosis co-occur during inflammatory responses, with ferroptosis triggering inflammation and necroptosis further exacerbating the release of pro-inflammatory factors. However, as to cuproptosis, elevated copper concentrations have been observed in tumor tissues and/or blood of various cancer patients, including those with breast cancer, lung cancer, and gastrointestinal cancers. The role of copper in cancer is complex, as it can both promote tumor development and potentially inhibit tumor growth. While copper is crucial for the proliferation of cancer cells, excessive copper influx can induce cell death, particularly through mitochondrial pathways. The increased availability of copper ion carriers facilitates the transport of copper ions across cell membranes, presenting novel methods for inducing cell death in cancer therapy. In terms of cuproptosis and disulfide cell death in tumor cells is as shown in Figure 13.

The occurrence of disulfide death requires the simultaneous fulfillment of two key conditions: Glucose depletion, and Cells are unable to generate sufficient reduced nicotinamide adenine dinucleotide phosphate (NADPH) through the pentose phosphate pathway (PPP), leading to insufficient reducing capacity.

High expression of SLC7A11: Solute carrier family 7 member 11 (SLC7A11) is a cystine transporter protein whose high expression promotes excessive cellular uptake of cystine. When glucose is abundant, NADPH can reduce cystine to cysteine, which is used to synthesize glutathione (GSH) to fight against oxidative stress. However, when glucose is deficient, the insufficient supply of NADPH leads to the inability to timely maintenance of redox homeostasis, resulting in the abnormal accumulation of disulfides (such as cystine) within cells, which triggers a stress response.

At the molecular mechanism level, disulfide stress: Excessive cystine within cells forms disulfide molecules, leading to abnormal cross-linking of disulfide bonds, particularly between cytoskeletal proteins such as actin.

Cytoskeletal contraction and collapse: Abnormal cross-linking of the actin network causes cytoskeletal contraction and detachment from the plasma membrane, ultimately disrupting cellular morphology and structural integrity, triggering rapid cell death.

Distinction from other forms of cell death: Disulfide death is independent of apoptosis, ferroptosis, necroptosis, and cuproptosis, with its specificity relying on disulfide bond cross-linking, differentiating it from lipid peroxidation in ferroptosis or copper ion toxicity in cuproptosis. The time from stress to death is relatively short (ranging from hours to a day), possibly related to acute disruption of the cytoskeleton.

Since its discovery in 2023, research on disulfide death has rapidly increased, with potential new therapeutic strategies targeting tumors with high SLC7A11 expression (such as lung cancer and breast cancer) through the inhibition of glucose metabolism or in combination with GLUT inhibitors. Disulfide death, centered around cystine metabolic imbalance, disrupts the cytoskeleton through disulfide bond cross-linking, providing new targets for tumor treatment. Research into its mechanisms is still in the early stages, and further exploration of its interaction with redox homeostasis and metabolic reprogramming, as well as the development of specific induction or inhibition strategies, is necessary in the future.

Stasis. It can also promote the production of interferon and increase T cell activation, while raising cAMP levels in tumor cells, thereby inhibiting their proliferation. Additionally, it inhibits sialidase activity and the formation of mutated cells. The combination of Astragalus membranaceus and Dioscorea opposita originates from Shi Jinmo Duoyao (A Collection of Medicines), and illustrates therapeutic effects.

### 4.8 Immunotherapy for cancer treatment

Astragalus membranaceus-Dioscorea opposita regulates the secretion of mucus in the respiratory, digestive, and urogenital tracts, while enhancing the body’s first line of immune defense. Moreover, Astragalus-Dioscorea polysaccharides promote the growth of beneficial microbiota in the intestines of broiler chickens and in conditions such as ulcerative colitis, playing a regulatory role in maintaining microecological balance. In network pharmacology, Zhao Jiahua et al. demonstrated through network pharmacological analysis that Astragalus membranaceus has a solid foundation for in-depth research and application, and regulates multiple targets and signaling pathways (Bai et al., 2024). Zhang Liang et al. used network pharmacology and molecular docking to explore the mechanism of Dioscorea opposita in treating gastric cancer (Wen et al., 2023). They identified 12 active components of Dioscorea opposita and 119 potential targets, with 56 common targets related to gastric cancer. Through further analysis, TP53, HSP90AA1, and AKT1 were identified as key targets for Dioscorea opposita in treating gastric cancer. Astragalus membranaceus-Dioscorea opposita contains trace elements that can inhibit cancer cell metastasis by tonifying the spleen, benefiting the kidneys, consolidating essence, and treating conditions like diabetes and edema. Modern pharmacological research has confirmed that both Astragalus membranaceus and Dioscorea opposita, as well as their compound formulations, have protective effects on diabetic nephropathy. The combination of two herbs further enhances its antioxidant capacity and provides kidney protection against diabetic nephropathy (Yang et al., 2009a). Contemporary pharmacological research indicates that each herb of Astragalus membranaceus-Dioscorea opposita has its unique actions. Astragalus membranaceus primarily enhances non-specific immune functions in the immune system (Jin, 2017), whereas Dioscorea opposita is effective in suppressing oxidative stress in the body (Hai et al., 2016; Tian et al., 2012; Yang et al., 2014). As a well-established herb pair in TCM, Astragalus membranaceus-Dioscorea opposita has been widely used for various medical conditions. Numerous studies have shown that Astragalus-Dioscorea exhibits extensive biological activities, such as anti-aging, anti-cancer, antioxidant, immune regulation, and anti-inflammatory effects. Therefore, it has been extensively used in the treatment of cardiovascular diseases, diabetes, cancer, and other conditions (Ko and Chik, 2009; Namuga et al., 2024; Zhang et al., 2023). Astragalus membranaceus contains complex chemical components, including Astragalus polysaccharides (APS), flavonoids, and astragaloside IV (AS-IV) (Choi et al., 2010; Guo et al., 2021; Jiang et al., 2017; Mrena, 2005; Wang et al., 2020).

**FIGURE 14:**
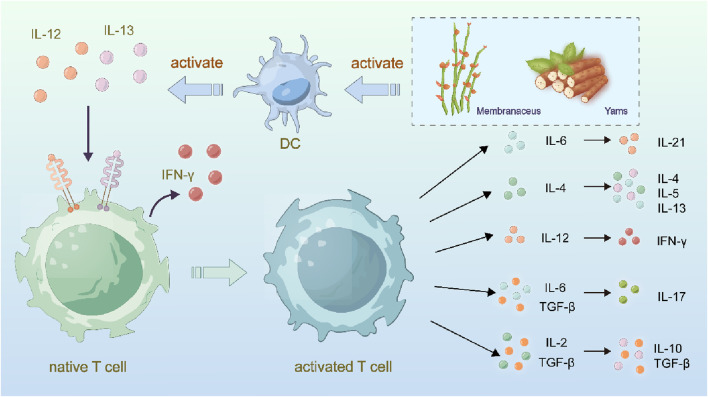
Anti-cancer mechanisms of Astragalus membranaceus-Dioscorea opposita in cellular immunity.

Notably, Astragalus polysaccharides, derived from Astragalus membranaceus, are the most significant active components, with pronounced immune-regulating properties (Bhattacharyya et al., 2017; Hwang et al., 2019; Li et al., 2017). Extensive *in vitro* and *in vivo* studies have confirmed that Astragalus polysaccharides can modulate the immune system. APS is an excellent immune enhancer for both humoral and cellular immunity (Li et al., 2017). APS accelerates the maturation of dendritic cells (DC), enhances their antigen-presenting capability, and reduces their phagocytic activity. Furthermore, APS can induce the differentiation of DC, which in turn activates T cells (Songtian et al., 2021). APS controls T cell immunity by binding to the Toll-like receptor 4 (TLR4) on Tregs, limiting the activity of CD4^+^CD25^+^ Tregs, and promoting the shift from Th2 to Th1 in CD4^+^ T cells. Additionally, APS can activate B cells independently of TLR4 via membrane immunoglobulin. Dioscorea opposita, first recorded in the Shennong Bencao Jing (Shennong Classic of Materia Medica), is an excellent food-medicine hybrid plant with a long cultivation and medicinal history. In May 2020, it was included in the National Health Commission’s traditional Chinese medicine rehabilitation plan for Coronavirus Disease 2019 (COVID-19) (Zou et al., 2006). Liu Xuan investigated the immune-modulating effects of sulfated Dioscorea polysaccharides on RAW264.7 macrophages through RNA-seq and developed a Dioscorea polysaccharide oral liquid. Kong Chenxian et al. conducted a meta-analysis on the immune-regulating effects of Dioscorea polysaccharides in animals, which was based on literature from various databases such as CNKI, VIP, Wanfang, PubMed, and Springer (Yi et al., 2007). The indirect comparison method was used to manually screen relevant literature by reviewing abstracts and discussions. Meta-analysis was performed on the selected studies using RevMan 5.3 software. The results showed that both Astragalus membranaceus and Dioscorea opposita are commonly used in immunotherapy for cancer treatment. Astragalus and Dioscorea opposita activate the immune system through different pathways. Astragalus emphasizes enhancing overall immune function, while Dioscorea opposita strengthens local immune responses. The combination of the two creates a more comprehensive immune defense network. Astragalus polysaccharides (APS) and astragalus saponins significantly enhance the body’s immune function by increasing T lymphocyte activity, promoting macrophage phagocytosis, and increasing the secretion of cytokines (such as IL-2 and TNF-α), thereby activating both innate and adaptive immune responses, which enhances the ability to recognize and kill tumor cells. Dioscorea opposita polysaccharides (such as RDPS-1) can activate macrophages and NK cells, boosting their phagocytic and cytotoxic abilities against tumor cells while also regulating the Th1/Th2 balance to promote anti-tumor immune responses. Furthermore, saponin components in Dioscorea opposita can induce apoptosis in tumor cells, further assisting the immune system in eliminating abnormal cells. Immunotherapy for Cancer Treatment is shown in Figure 14.

**FIGURE 15:**
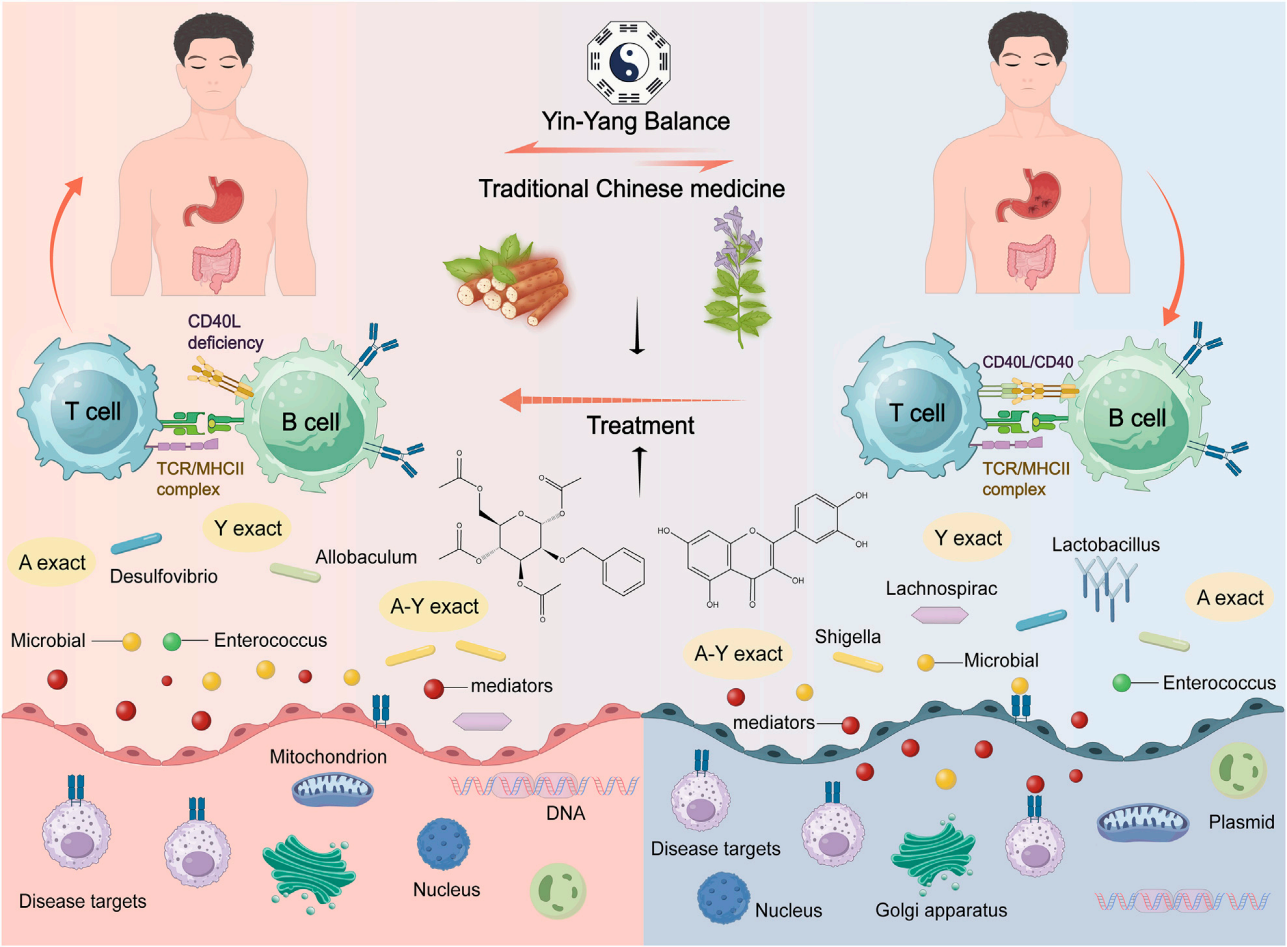
Mechanism diagram of Astragalus and Dioscorea opposita gut microbiota.

Astragalus and Dioscorea opposita, as traditional Chinese medicines, have demonstrated potential value in regulating gut microbiota and assisting in cancer treatment in recent years. Astragalus is rich in active components such as polysaccharides, flavonoids, and saponins, which can enhance gut barrier function, modulate immune responses, promote the proliferation of beneficial bacteria (such as *Lactobacillus* and Bifidobacterium), and inhibit the growth of pathogenic bacteria, thus improving the dysbiosis of gut microecology. The mucoproteins, diosgenin, and dietary fibers in Dioscorea opposita selectively nourish beneficial intestinal microbiota (Zhou et al., 2022), promote the generation of short-chain fatty acids (such as butyrate), and regulate the body’s anti-tumor immune activity through the gut-immune axis (Cui et al., 2023). Research indicates that their synergistic effects may reshape the structure of gut microbiota, reduce the expression of pro-inflammatory factors (such as IL-6 and TNF-α), activate anti-cancer signaling pathways (such as PI3K/AKT/mTOR), inhibit tumor cell proliferation, and enhance the sensitivity to chemotherapeutic drugs, providing a “microbiota-immune-metabolism” multi-target intervention strategy for adjuvant cancer therapy. The gut microbiome mechanism is shown in Figure 15.

## 5 Clinical research

As traditional Chinese medicines, Astragalus membranaceus and Dioscorea opposita have shown multi-target and multi-pathway synergistic anti-tumor effects in cancer clinical research. Astragalus plays a central role by enhancing the body’s immune function, and its polysaccharide components can regulate Th1/Th2 cell balance, inhibit pro-inflammatory factors such as IL-4 and IL-6, and promote the secretion of IL-2, TNF-α and IFN-γ, and enhance the anti-tumor activity of CD8^+^ T cells (Qiang et al., 2012). Clinical research shows that Astragalus injection can significantly improve the immune function of chemotherapy patients, reduce bone marrow suppression, and enhance the efficacy of chemotherapy drugs such as cisplatin. In addition, flavonoid components of Astragalus downregulate the expression of miR-582-3p, relieving its inhibition on p27, thereby blocking the cell cycle of lung adenocarcinoma cells and inducing apoptosis (Auyeung et al., 2012). Dioscorea opposita regulates tumor-related molecular axes through active polysaccharides and trace elements such as germanium. Research has found that Dioscorea opposita polysaccharide (CYPS) can downregulate the expression of miR-98-5p in liver cancer cells (Zheng et al., 2021). It promotes the expression of TGFβR1, thereby activating apoptotic proteins such as Caspase-3 and Caspase-8, which inhibit the proliferation of Huh-7 liver cancer cells and induce apoptosis. Its immunomodulatory effects are also reflected in promoting the generation of interferon and T cell proliferation, while inhibiting the cAMP levels in tumor cells, thus limiting cancer cell metastasis. In clinical applications, Dioscorea opposita is often combined with astragalus to synergistically regulate the IL-17 and TNF signaling pathways, suppressing the inflammatory response in the tumor microenvironment and indirectly inhibiting tumor growth (Wang et al., 2007).

**FIGURE 16:**
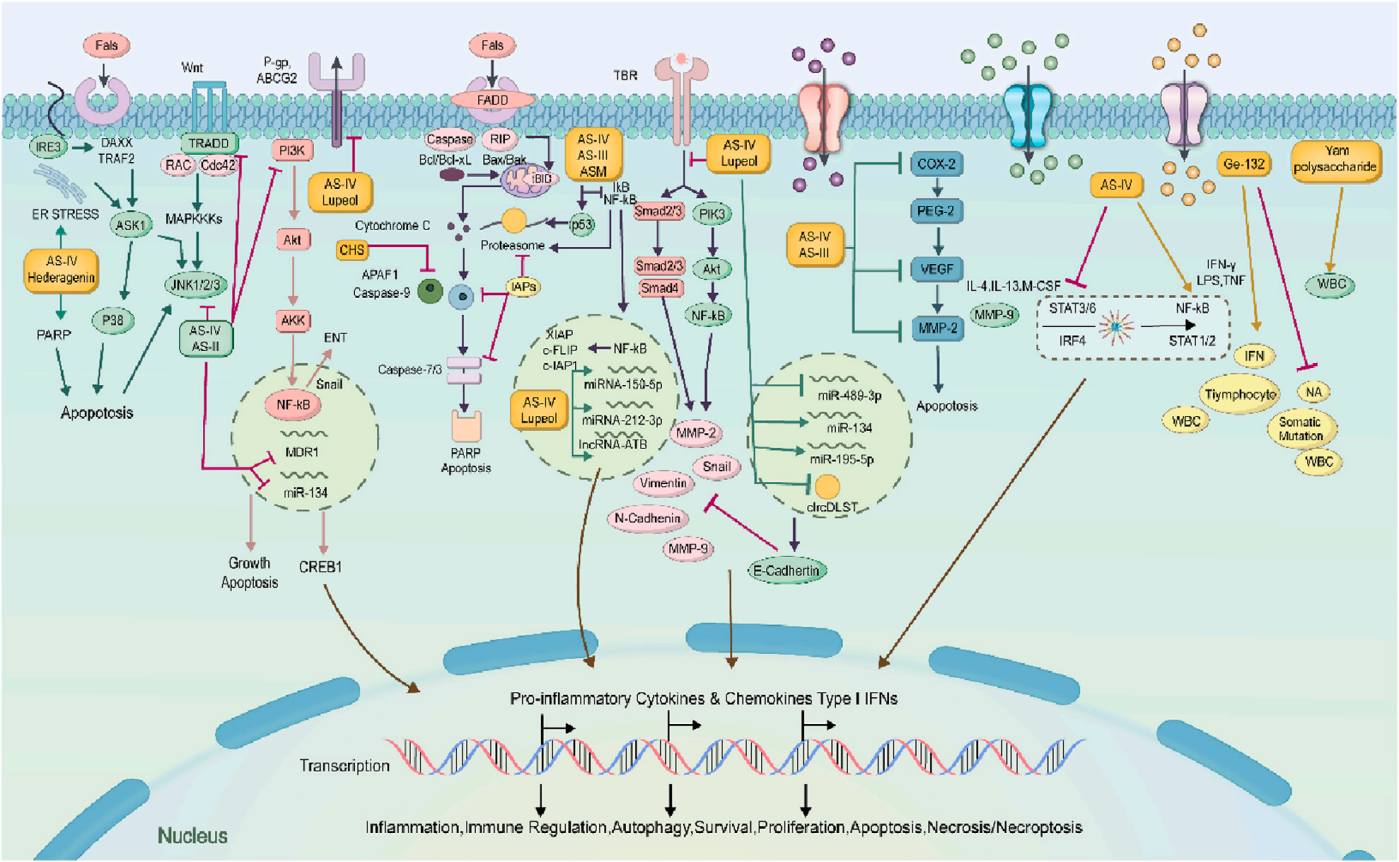
Mechanisms of anti-cancer effects of Astragalus membranaceus-Dioscorea opposita and its active ingredients.

**FIGURE 17:**
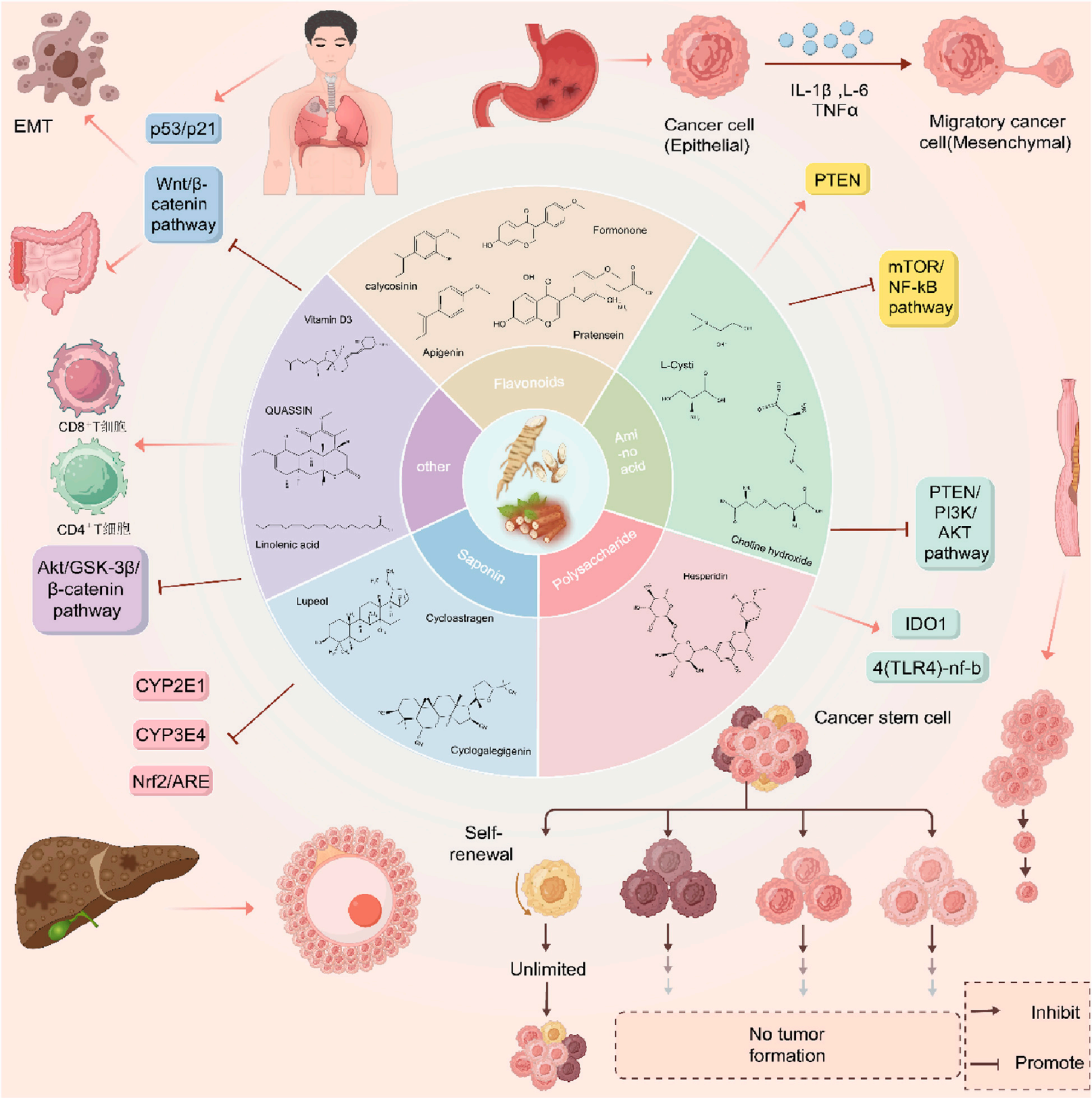
Mechanisms of anti-cancer effects of Astragalus membranaceus-Dioscorea opposita and its active ingredients.

The combined application of both has also shown potential in reversing chemotherapy resistance. Astragalus improves the hypoxic state of tumors by inhibiting P-glycoprotein (P-gp) and reducing the level of HO-1, while Dioscorea opposita polysaccharides may enhance the toxicity of chemotherapy drugs by regulating glutathione metabolism. Although many existing studies are based on cellular and animal experiments, several clinical trials have confirmed their synergistic effects in reducing toxicity and enhancing efficacy. For example, the combination of Astragalus and chemotherapy can decrease adverse reactions such as nausea and vomiting in patients and improve their quality of life. Further exploration of dose-dependent effects and synergistic mechanisms with other targeted drugs is needed to promote clinical translation. Astragaloside IV, a major active component in Astragalus membranaceus, has been demonstrated to inhibit the activity of key subtypes of the cytochrome P450 (CYP450) enzyme system (e.g., CYP3A4, CYP2D6) in human liver microsomes (Li et al., 2024). Notably, commonly used chemotherapeutic agents such as paclitaxel, docetaxel, and irinotecan are predominantly metabolized by CYP3A4. Consequently, co-administration of astragaloside IV with these chemotherapeutics may lead to a reduction in the plasma clearance rate of chemotherapeutics and an elevation in their plasma concentrations. This, in turn, significantly increases the risk of adverse events, including myelosuppression (e.g., a 15%–20% increase in the incidence of grade Ⅳ neutropenia) and neurotoxicity (e.g., exacerbation of paclitaxel-associated peripheral neuropathy). Conversely, polysaccharides derived from Dioscorea opposita can induce the expression of UDP-glucuronosyltransferases (UGTs) (Liu et al., 2022). This induction accelerates the clearance of drugs that rely on UGT-mediated metabolism (e.g., capecitabine), potentially resulting in insufficient therapeutic exposure to the chemotherapeutic agent and thereby impairing its antitumor efficacy. The overall mechanism of astragalus and Dioscorea opposita is shown in Figures 16, 17.

## 6 Conclusion and outlook

Traditional Chinese medicine (TCM) has a long developmental history and plays an important role in the prevention and treatment of malignant tumors. It possesses a unique holistic perspective and dialectical methodology that distinguishes it from Western medicine, offering a different viewpoint in disease management. According to TCM theory, diseases arise from an imbalance of yin and yang, and the goal of TCM is to restore this balance to alleviate the symptoms of illness. This holistic approach not only directly targets tumors but also considers the overall health of the patient. TCM regards cancer as a reaction to the disorder of qi and blood circulation and the accumulation of phlegm and turbidity within the body. It emphasizes the importance of restoring harmony in bodily functions and mental health for healing, which aligns closely with modern medicine’s focus on addressing patients’ psychological and emotional wellbeing in cancer treatment. The therapeutic effects of these herbs are related to their capabilities in SBR, CHT, EDRP, and PBCRBS. Among them, herbs with SBR and CHT functions and CMF are most commonly used in treating IC. The molecular mechanisms underlying the anti-IC effects relate to the inhibition of cell proliferation, metastasis, and angiogenesis, induction of apoptosis, reversal of chemoresistance, and modulation of immune responses. These herbs and their components, along with CMF, regulate various pathways to exert their anti-IC effects, such as MAPK, NF-κB, PI3K/AKT, and EMT. An analysis of 152 studies (n > 65,000) has shown that the utilization rate of complementary and alternative medicine (CAM) among cancer patients has increased from 32% (before the 2000s) to 49% in recent years. Among these CAM modalities, Traditional Chinese Medicine (TCM) – including herbs such as Astragalus membranaceus (Huangqi) and Ganoderma lucidum (Lingzhi) – accounts for the largest proportion (38%). Importantly, TCM use is significantly associated with prolonged overall survival in cancer patients [hazard ratio (HR) = 0.82, 95% confidence interval (CI): 0.71–0.95] (CACA Guidelines Writing Committee, 2025).

Compared to other chemotherapy drugs used in Western medicine for the treatment of colon cancer, traditional Chinese medicine (TCM) has garnered significant attention in recent decades as a potential therapeutic option due to its multi-component, multi-target, and multi-pathway characteristics. TCM is known for having minimal side effects, the ability to enhance patients’ immune systems, and improving the quality of life during and after treatment. Furthermore, the high costs of chemotherapy and targeted therapy obstruct their widespread acceptance among patients in developing countries, making the relatively lower cost of TCM a distinct advantage for many patients around the world. In contrast, Western medicine typically targets cancer cells directly through standardized treatments such as surgery, chemotherapy, and radiation therapy. Given the fundamental differences in how Western and traditional Chinese medicine approach disease treatment, randomized controlled trials (RCTs) based on Western medical diagnoses may not be the most appropriate method for assessing the efficacy of TCM. Additionally, there are practical challenges in conducting clinical trials for TCM, as it does not adhere to the standardized “one-size-fits-all” approach of Western medicine; rather, TCM formulations are often customized according to individual needs. Therefore, employing pragmatic trial designs may be more suitable for evaluating TCM. In this regard, the principles of TCM appear to align with the contemporary concept of precision medicine utilized in oncology.CHM offers a unique and comprehensive approach to managing IC, with the potential to improve patient treatment outcomes and quality of life. However, most of the chemical preventive effects of these herbs have been studied in various human cancer cell lines, with less research conducted in animal tumor models. Challenges such as the standardization of CHM formulations and the design of rigorous clinical trials still exist. Further research is crucial for evaluating the therapeutic effects of CHM on IC. More clinical trials and cohort studies are needed to determine the therapeutic benefits of these herbs. The future research of integrative oncology focuses on three core areas: In clinical trials, conduct multicenter randomized controlled trials of integrated Traditional Chinese Medicine and Western medicine, as well as biomarker-guided adaptive designs to optimize intervention regimens; mechanistic research centers on deciphering tumor microenvironment regulation and metabolic reprogramming via single-cell multi-omics; simultaneously develop gut microbiota, metabolite, and molecular imaging biomarkers. It is also necessary to strengthen TCM quality control to ultimately achieve precise integration and improve the efficacy and safety of cancer treatment, and pay attention to adverse reactions and toxicity grading. And be mindful of the risk of bias, The visual summary of risk of bias (traffic light plot) serves as the “fundamental language” of a comprehensive analysis, while the discussion on the impact of high-risk studies acts as the “guarantee for the reliability of conclusions” — only the combination of the two can make the comprehensive analysis both intuitive and rigorous, truly providing references for clinical practice and subsequent research.
